# Gut Immune System and the Implications of Oral-Administered Immunoprophylaxis in Finfish Aquaculture

**DOI:** 10.3389/fimmu.2021.773193

**Published:** 2021-12-16

**Authors:** Po-Tsang Lee, Fernando Y. Yamamoto, Chen-Fei Low, Jiun-Yan Loh, Chou-Min Chong

**Affiliations:** ^1^ Department of Aquaculture, National Taiwan Ocean University, Keelung, Taiwan; ^2^ Thad Cochran National Warmwater Aquaculture Center, Mississippi Agriculture and Forestry Experiment Station, Mississippi State University, Stoneville, MS, United States; ^3^ Institute of Systems Biology, Universiti Kebangsaan Malaysia, Bangi, Malaysia; ^4^ Centre of Research for Advanced Aquaculture (CORAA), UCSI University, Cheras, Malaysia; ^5^ Aquatic Animal Health and Therapeutics Laboratory (AquaHealth), Institute of Bioscience, Universiti Putra Malaysia, Serdang, Malaysia

**Keywords:** GALT, gut immunity, immunostimulants, immunoprophylaxis, immune tolerance

## Abstract

The gastrointestinal immune system plays an important role in immune homeostasis regulation. It regulates the symbiotic host-microbiome interactions by training and developing the host’s innate and adaptive immunity. This interaction plays a vital role in host defence mechanisms and at the same time, balancing the endogenous perturbations of the host immune homeostasis. The fish gastrointestinal immune system is armed with intricate diffused gut-associated lymphoid tissues (GALTs) that establish tolerance toward the enormous commensal gut microbiome while preserving immune responses against the intrusion of enteric pathogens. A comprehensive understanding of the intestinal immune system is a prerequisite for developing an oral vaccine and immunostimulants in aquaculture, particularly in cultured fish species. In this review, we outline the remarkable features of gut immunity and the essential components of gut-associated lymphoid tissue. The mechanistic principles underlying the antigen absorption and uptake through the intestinal epithelial, and the subsequent immune activation through a series of molecular events are reviewed. The emphasis is on the significance of gut immunity in oral administration of immunoprophylactics, and the different potential adjuvants that circumvent intestinal immune tolerance. Comprehension of the intestinal immune system is pivotal for developing effective fish vaccines that can be delivered orally, which is less labour-intensive and could improve fish health and facilitate disease management in the aquaculture industry.

## Introduction

Diseases have always been the “Achilles’ heel” of intensive farming, and that analogy is especially accurate for aquaculture. With the development of technologies and intensification of production, several sources of stress such as animal handling, poor water quality, and overcrowding can compromise the fish immune system (1), and the water of enclosed intensive system can serve as a medium that facilitates the horizontal transmission of pathogens in the cultured species (2). Farmed fish are constantly exposed to opportunistic pathogens that naturally inhabit the nutrient-rich culture water (3). As it is connected to the external environment, the gastrointestinal tract is considered one of the main sites of pathogen translocation (4, 5). The gastrointestinal tract is a multifaceted system with several roles that go beyond the absorption of nutrients (5). The recent advances unravel the intricate interactions between the intestinal microbiome, dietary intake, and gut local immune system, and in turn how it affects the host physiological responses and health. And the impairment of gut health has been directly linked to the rising susceptibility to enteric infections (6, 7).

Aquaculture is the fastest-growing animal production industry and it leverages on practicability and profitability (8). Owing to high practicability and stress-free administration, oral supplements and vaccines that can ameliorate fish health has been gaining traction and popularity in research, and understanding of gut immune system paves the way to the development of the oral application. Researchers and farmers of fed-aquaculture (*i.e.*, fish that rely on nutrient input from formulated diets) are allowed to manipulate the health of the finfish through the diets by either incorporating feed additives, drugs, or even vaccines (5). Several feed supplements have long been investigated (*e.g.*, immunostimulants, immunonutrients, phytotherapeutics, etc.) and have been reported to enhance the intestinal health of an array of aquatic species. The uniqueness of how each of these orally administered interventions could ameliorate the physiological responses of the host, or how their mechanisms can inhibit pathogen proliferation, deserves to be appraised in a holistic view with the fish intestinal immune system, which ultimately prevents disease outbreaks and economic losses. In this review, a compilation of recent studies on intestinal immunology of farmed finfish will be interpreted and discussed, as well as the advent of orally delivered vaccines, and plant-based immunostimulants on fish intestinal health.

## Gut Immunity

### Regionalization of Gastrointestinal Tract

The gastrointestinal tract is a hollow muscular tube that connects a series of alimentary organs, starting at the buccal cavity to the rectum (9). It is a multifunctional system that is not restricted only to digestion, but also nutrient absorption and sensing, water and electrolyte balance, hormone secretion, and the more challenging task to establish immunity (10). Although teleosts exhibit great heterogeneity in terms of morpho-histology of the gastrointestinal tract (11), the gut structure can be separated into three segments (12, 13).

The first segment, commonly known as the foregut or anterior gut, is a topographical region of the gastrointestinal tract where the chemical digestion of ingested food matters begins (14). In this segment, the absorption of dietary protein takes place by enterocytes or intestinal absorptive cells (13). As the longest portion of the gut (9) and the site for the majority of digestive activities (15), the second segment or the midgut possesses enzymes from the pancreas, liver and intestinal wall to catalyze digestion and uptake of macromolecules (16). The midgut harbors highly diverse microbial consortia that are believed to take part in the digestion (17). Commonly termed as the hindgut, the third segment of the gastrointestinal tract is engaged in osmoregulatory activities such as ion transport and water reabsorption (13). Some studies described the teleost hindgut as homologues to the mammalian large intestine (18). As nutrient uptake progressively reduces (14) along the intestinal tract, the importance of immune homeostasis mechanisms gradually increases from the foregut to the hindgut segments (19). These trends have been supported by the higher transcript levels of immune-related genes along with the appearance of smaller irregular intestinal folds from the foregut to the hindgut (20, 21).

Unlike mammals, separations or transitions between the different functional segments of the teleost gut are not clearly defined (18). The trichotomous division of the teleost gut into segments is obscure and inconsistent in research. For instance, some studies defined the proximal region of the intestine immediately after the stomach and pyloric ceca as the foregut (22–25), whereas others considered the stomach as the foregut (9, 26).

It should be noted that there are studies that have categorized the entire gut into two segments instead of three (fore, mid and hind); therefore, the third gut segment was neglected or not recognized in these studies (9, 12). Some studies conducted with Atlantic salmon (*Salmo salar*) had divided the intestine into the mid and posterior regions, where the mid-intestine region was further subdivided into two, termed the first and second segments of the midgut (18, 20, 27).

For the agastric fish, the entire gut (after the oesophagus) is divided into segments of equal length, *e.g.*, 4 equal segments in ballan wrasse (*Labrus bergylta*) and 7 equal segments in zebrafish (*Danio rerio*). Segment 1, segments 2 - 3, and segment 4, represent the foregut, midgut, and hindgut of ballan wrasse, respectively (28). In zebrafish, segments 1-5 exhibits foregut/midgut features, whereas the segment 6 and 7 behave like the hindgut (19).

### Gut-Associated Lymphoid Tissue

As one of the major parts of mucosal lymphoid tissues that are constantly connected with external environment and could be primary entry sites of pathogenic intrusions, fish gut-associated lymphoid tissues (GALTs) play an indispensable role in fish health (29). Unlike mammalian GALTs that possess organized structures such as mesenteric lymph nodes and Peyer’s patches (9), teleosts have diffusely organized GALTs that accommodate abundant myeloid and lymphoid cells, which regulate homeostasis to protect the host from potentially pathogenic microbes and to tolerate anodyne food-derived antigens and commensal microbiota (9, 30).

All segments of the gastrointestinal tract comprise four concentric layers. From the outer lining of the gut inwards, these layers are: the serosa, the outermost of the gut that consists of a thin coating of squamous epithelium and connective tissue; the muscularis, a deeper layer consisting of muscle fibre sheets; the submucosa, might be absent completely in some teleost species such as zebrafish, is a concentric layer that is made up of loose connective tissues; and the mucosa, the innermost layer that acts as a physical-chemical barrier towards intestinal lining, and it is where the diffusely organised GALTs reside (9, 18, 31). Unlike the mammalian mucosa, the finger-like mucosal villi are absent in the teleost intestinal lining. The fish intestinal lining possesses intestinal folds that consist of a monolayer of columnar epithelium on the microvilli’s cell surface (18).

The gut epithelial layer acts as an intrinsic physical layer that defends the host from the invasion of the harmful antigens (5). The epithelium expresses a wide spectrum of bioactive soluble factors, such as antimicrobial peptides, signaling molecules and toxin-neutralizing enzymes such as alkaline phosphatase. These defense molecules are also present in the mucus produced by intraepithelial goblet cells (32). Moreover, mucus harbors a high volume of mucins that cause the viscous nature of mucus. This glycoprotein dissociates microbes and impedes microbial adherence to the fish mucosa. Together with the epithelial layer, the extrinsic barrier conferred by the mucus protects the GALTs from the hostile environment of the gastrointestinal tract (9).

**Figure 1:**
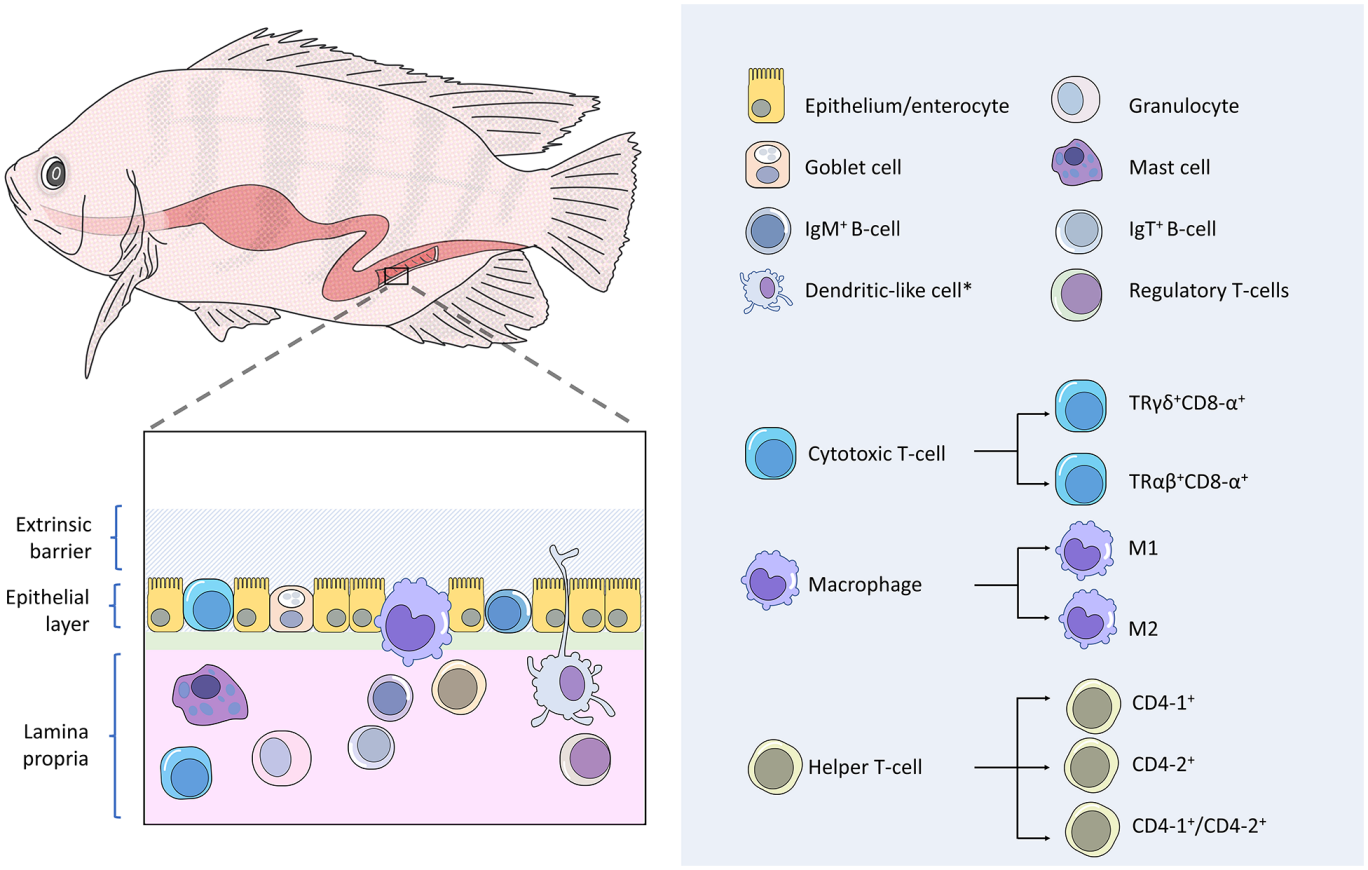
Gut-associated lymphoid tissues (GALT) of finfish. Fish gut possesses diffusely organized GALTs that comprise two main leukocyte populations. Firstly, intraepithelial leukocytes, which mostly consisted of IgM+ B-cells, cytotoxic T-cells, and macrophages. Secondly, the lamina propria leukocytes, which are consisting of lymphocytes, macrophages, granulocytes and dendritic-like cells. Subpopulations have been reported in teleost cytotoxic T-cells, macrophages, and helper T-cells.

The GALTs (**Figure 1**) comprise two main leukocyte populations: (1) intraepithelial lymphocytes (IELs), which refer to the adaptive lymphoid cells that reside in the epithelial layer; (2) lamina propria leukocytes, which are consisting of lymphocytes, macrophages, granulocytes and dendritic-like cells (12, 33). The IgT^+^ B-cells have been reported as the predominant IELs in carp and sea bass (13, 34), whereas the IELs are primarily CD8-α^+^ cells (Cytotoxic T-cells) in sea bass (35).

The IgT^+^ B-cells release antibodies of the IgT isotype, which acts as mucosal-associated antibodies to coat teleost luminal microbiota, analogous to mammalian IgA and amphibian IgX (34). IgT appears to be the ortholog of IgZ as these molecules are similar in structure and genome information (36). Two subclasses of IgZ have been identified in zebrafish, namely IgZ and IgZ2 (37). Functional analysis revealed that the IgZ antibodies activate complement-mediated lysis and the IgZ2 antibodies are unable to trigger complement activity but can coat and neutralize the microbes of the zebrafish gut, in turn, prevent microbial translocation across the epithelium (36).

The CD8-α^+^ cells are another IEL in the fish gut. The cluster of differentiation 8 alpha (CD8-α) is a receptor expressed by the teleost cytotoxic T-cells. In addition to CD8-α, teleost intraepithelial cytotoxic T-cells bear T-cell receptors of the γδ heterodimers (TRγδ) that act like pattern recognition receptors (PRRs) to recognize antigen in a more non-specific manner in contrast to the systemic CD8-α^+^ cells that express αβ T-cell receptors (TRαβ) (38). The latter binds to major histocompatibility complex (MHC)-bound antigens specifically (39). Based on a molecular study that showed the gene expression of *mhc-Iα* and *cd8-α* of sea bass (*Dicentrarchus labrax*) intestine were not correlated, it has been postulated that fish intraepithelial CD8^+^ cells are able to recognize antigens without association with MHC molecules (35, 38). The innate immune features continue to be found on the teleost γδ T cells as a study of zebrafish (40) demonstrated that these IELs are capable of performing non-specific phagocytosis toward both particulate and soluble antigens, and in turn act as antigen-presenting cells to activate adaptive immunity, including IgZ B-cells.

In addition to IELs, macrophages have been reported to be in epithelial compartments of some teleost species (38). Adjacent to the intestinal lumen, the epithelium-associated macrophages are specialized in scavenging and phagocytosing apoptotic epithelial cells and potentially harmful microbes (41). Mucosal macrophages also reside in the teleost lamina propria and intestinal muscularis. While the lamina propria macrophages function as antigen-presenting cells and cytokine producers to moderate local immune protections, the recently discovered muscularis macrophages are highly innervated and may play motility-oriented and neuroprotective roles in the teleost enteric nervous system, albeit their functional significance across multiple layers of the muscularis remain not clear (41). A study on zebrafish showed that mucosal macrophages can shape the intestinal microbial composition through the expression of interferon regulatory factor *irf8* (42). An ineffective complement system followed by severe dysregulation of commensal microbiota was found in the adult *irf8*-deficient zebrafish. Akin to the mammalian macrophage, the bony fish have two subsets of macrophages: proinflammatory macrophages termed as M1 that produces TNF-α, and anti-inflammatory macrophages named as M2 and they are inept at producing TNF-α (43). These two macrophage subgroups are differentiated and polarized by distinct microenvironmental factors, where M1 is committed to the establishment of the inflammatory process and M2 is involved in the repair activity during the resolution phase of inflammation (44).

Within the lamina propria, which is the underlying immune-rich connective tissue beneath the epithelial layer, tissue granulocytes can be found disseminated throughout the teleost gut. Similar to other phagocytic cells such as macrophages and dendritic-like cells, teleost granulocytes carry pattern recognition receptors to sense the presence of intruding pathogens through microbe-associated molecular patterns (32). Upon detection of a pathogen, immune protective responses are immediately taken place by the granulocytes through phagocytosis. The activated granulocytes will then produce histamine and chemokines that promote vasodilation of intestinal blood vessels and leukocyte infiltration (38). To protect the host from pathogens, neutrophils, which are a subset of fish granulocytes, release a vast array of bactericidal molecules such as antimicrobial peptides, peroxidase enzymes, reactive oxygen and nitrogen intermediates (45). Neutrophils are among the granulocytes that can readily infiltrate the teleost epithelium despite their low abundance in the teleost gut (38). Mast cells, also known as eosinophilic granular cells, are the granulocyte subpopulation that is functionally analogous to mammalian mast cells and highly vigilant against microbial intrusion through the fish mucosal barrier (18). Upon activation by sensing pathogenic invasion, teleost mast cells perform cell degranulation (38) and produce mediators of inflammation (46). Teleost mast cells have been found to mobilize and degranulate in response to parasitic infections in the gut (47).

Teleost lamina propria houses a great diversity of cellular components of the adaptive immunity necessary for a local defensive response. All the aforementioned lymphocytes that exist in the epithelium as IELs, namely the IgT^+^ B-cells and cytotoxic T-cells, can also be found in the teleost lamina propria (13, 38). In addition to these cells, the other lymphocyte subsets have been reported in the lamina propria of the bony fish, including IgM^+^ B-cells, IgD^+^ B-cells, CD8α^+^ cytotoxic T-cells, helper T-cells, and regulatory T-cells (9, 48). Hitherto studies have revealed that teleost fish cannot perform immunoglobulin class switching (49). All B-cells can express two forms of immunoglobulins: (1) B-cell receptor, which is a membrane-bound immunoglobulin that serves as a receptor for specific pathogen targeting; (2) antibody, which is the secreted immunoglobulin form that is produced by activated and differentiated B-cells, known as plasma cell or plasmablasts (13). The activated B cells can migrate in the gastrointestinal epithelial layer after antigenic stimulation (34, 50). The phagocytic ability of B-cells has been reported in some species (51, 52). Teleost B-cells can express three heavy immunoglobulin chain isotypes, *i.e.*, IgM, IgD, and IgT/IgZ (13), encoded by genes *μ*, *δ*, and *τ/ζ*, respectively (53). Although the function of IgT/Z antibodies has been elucidated elsewhere in the review, IgM antibodies that were previously perceived as the systemic adaptive humoral defense, have been found to coat mucosal resident microbes, albeit at a lower rate than the IgT antibodies (13). The binding of IgM antibodies to the corresponding targets initiates the complement-mediated lysis *via* the classical pathway, opsonization and agglutination of pathogens that facilitate pathogen clearance by phagocytes, blocking off the microbial active site, as well as neutralization of pathogen-derived toxin (54). The IgD B-cells are still an enigmatic B-lymphocyte subset as many relevant studies appear inconsistent in terms of cell lineage and function. For instance, rainbow trout IgM^+^ cells were found to co-express the IgD heavy chain (52, 55), but a unique IgM^-^/IgD^+^ B-cell population was reported in rainbow trout (*Oncorhynchus mykiss*) (56) and channel catfish (*Ictalurus punctatus*) (57). Functional studies have postulated that teleost IgD may be involved in mucosal homeostasis (55, 58, 59) as this immunoglobulin is produced to coat some gastrointestinal commensal bacteria (55, 59). However, rainbow trout IgD has been reported to be uninvolved in specific immunity in the mucosal organs during parasitic infestation (13). Due to divergence in findings, the immune function of teleost IgD remains mostly unclear and still inconclusive (13).

Unlike the intraepithelial layer that bears only CD8-α^+^ γδ cytotoxic T-cells, the teleost gut lamina propria possesses both the CD8-α^+^ T-cell populations of TRγδ and TRαβ (38, 60). CD8-α^+^ cells of TRαβ bind to intracellular pathogen-derived antigens that are presented by the MHC class I molecules expressed on the target cell and differentiate into activated effector cells to lyse the target cells (60).

CD4^+^ helper T-cells can be found in the teleost gastrointestinal lamina propria. This cell expresses T cell receptors and the cluster of differentiation 4 (CD4) on its surface for specific antigen recognition. In most reported teleost species, in exception to the gadoid line, antigen-presenting cells present antigenic peptides *via* the MHC-II and activate naive helper T-cells to make them proliferate into effector cells that produce inflammatory cytokines (32). These antigen-presenting cells include macrophages, granulocytes, dendritic-like cells as well as B-cells, and they release co-stimulatory molecules to prime helper T-cells (18, 32). Activated helper T-cells generate a cytokine cascade to coordinate and enhance host immune responses. These cells release IFN-γ to mediate teleost cellular defense, which involves the enhancements of CD8^+^ cell-mediated cytotoxicity and phagocytosis by macrophages (61). Humoral-mediated immunity can be elevated by teleost helper T-cells by regulating B-cell immune response (62). To date, few major types of helper T-cells have been reported in fish, namely CD4-1^+^ single-positive cells, CD4-2^+^ single-positive cells (63) as well as CD4-1^+^/CD4-2^+^ double-positive cells (44). And two subtypes of CD4-2, *i.e.*, CD4-2a and CD4-2b, have been reported in brown trout (64). Expression levels of CD4-1, CD4-2a, and CD4-2b were recorded to be different in the same tissues of rainbow trout against antigenic stimulation (64, 65). While CD4-1^+^ cells have been revealed to play a pivotal role against viral diseases in ginbuna crucian carp (*Carassius auratus*) (62), a study using olive flounder as the animal model showed that CD4-2 helper T-cells proliferated earlier and higher in number than CD4-1 cells (66), suggesting that CD4-2 cells are important in the early phase of cell-mediated immunity.

Regulatory T-cells are a subset of CD4-1^+^ cells in zebrafish (44, 67). A study on zebrafish (67) uncovered that the gut-derived CD4-1^+^ cells that were expressing the *foxp3a* and *il-10* genes, which are gene signatures for the regulatory T-cells. Studies have defined *foxp3a^+^* cells as the regulatory T-cells in fish due to the notion that the *foxp3a* is a regulatory factor involved in the immunosuppressive machinery, which includes suppressing cell proliferation and cytokine production of leukocytes (68, 69). Fish regulatory T-cells, known as *foxp3a^+^* cells, promote an anti-inflammatory response that restrains the over-exhaustive activities toward the mucosal non-self-antigens during steady-state, preventing an autoimmune disorder, and mobilizing to the damaged region to release tissue-specific regenerative factors that stimulate the proliferation of regeneration precursor cells (69).

### Absorption and Uptake of Antigens

Two major pathways of antigen uptake through the intraepithelial layer have been described in the mammal, *i.e.*, the paracellular and transcellular routes. The paracellular pathway refers to the rate-limited passive transport of inert or mostly cationic antigens, typically smaller than 600 daltons, through the tight junctions between the epithelial resident cells (18). Although it has been postulated that exogenous antigens can cross the fish epithelium through this route in the steady-state condition, to date, no study provides conclusive evidence to this hypothesis (18, 38).

**Figure 2:**
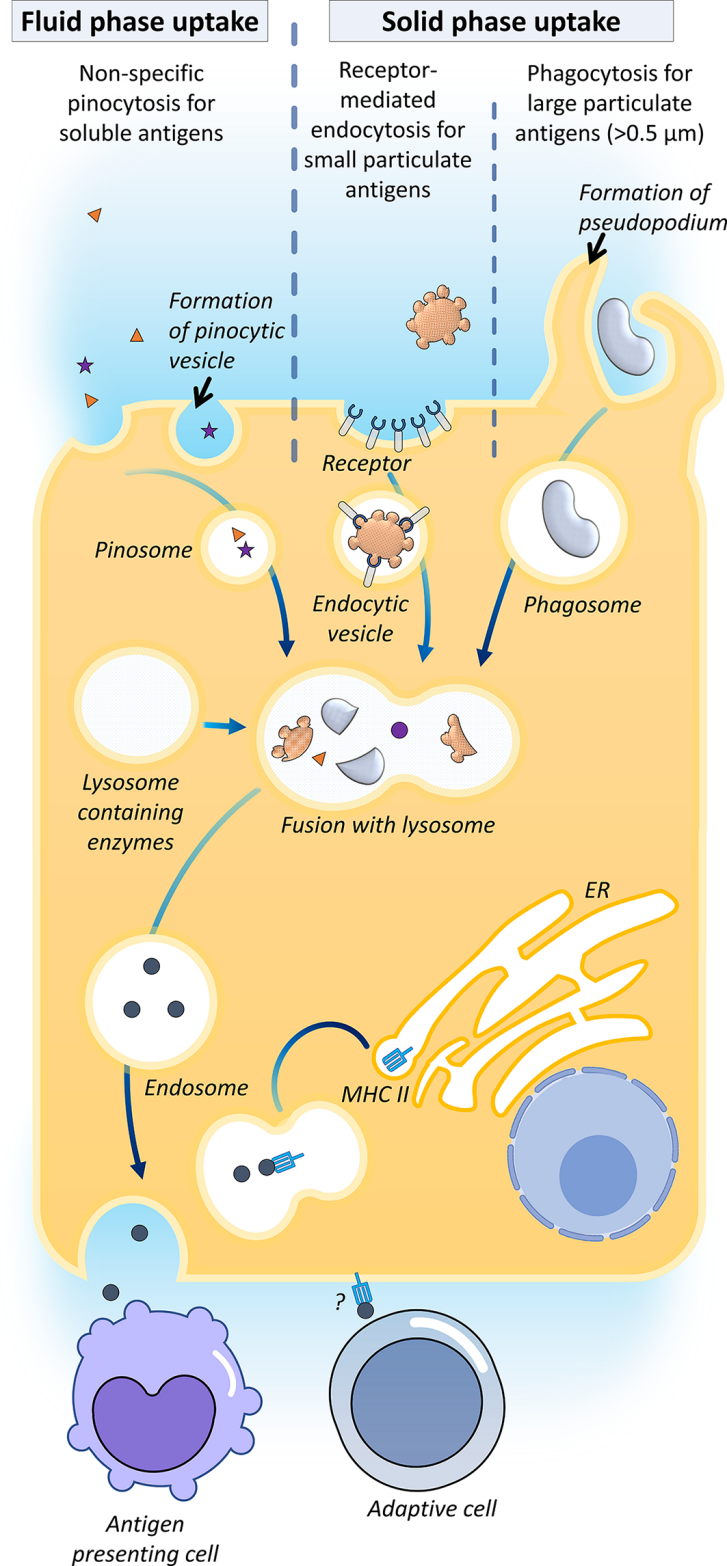
Antigen uptake in the gut. Their mechanisms of transcellular transport for exogenous antigens across teleost epithelial barriers have been described, namely fluid-phase uptake of soluble antigens by enterocytes *via* non-specific pinocytosis in carp and rainbow trout; solid-phase uptake of small solid antigens (<0.5 μm) by receptor-mediated endocytosis, and phagocytosis of larger particulate antigens. It has been proposed that the internalized antigens are processed in the endosome by merging with lysosomes containing enzymes, followed by systemic vascular release and transfer of the processed antigens to the intraepithelial or lamina propria antigen-presenting cells, viz. macrophages, some fish enterocytes expressed MHC-IIβ, which indicates that this cell might directly be serving as an antigen-presenting cell to activate the adaptive cells (35).

The transcellular transport of exogenous antigen across teleost epithelial barriers depends on the physical nature of the antigen (**Figure 2**). Fluid phase uptake of soluble antigens such as ferritin has been proven to take place *via* non-specific pinocytosis in grass carp (*Ctenopharyngodon idella*) and rainbow trout (38, 70–72). For small solid particles (<0.5 μm), as exemplified by HRP, solid-phase uptake by receptor-mediated endocytosis has been reported in the fish gut (18, 73). For larger particulate antigens, uptake can occur *via* phagocytosis, where the antigens are surrounded and internalized by the protrusion of cell membrane forming phagosomes (18, 74).

In higher vertebrates, the transcellular route can be accomplished by epithelium M-cells, dendritic cells and goblet cells (18). Most of the bony fish studies evinced that the luminal antigen uptake was attributed to the regular enterocyte, which is also known as the epithelial cell, with antigen absorptive ability (12, 18). In enterocytes, antigens are internalized into large supranuclear vacuoles (endosomes) merged with lysosomes containing enzymes, followed by systemic vascular release and transfer of processed antigens to intraepithelial or lamina propria macrophages (38, 71, 75). It has been proposed that the mechanism of this transfer is akin to the mammalian melanin transfer process in the retina (12). To date, the mechanism of the antigen transfer is yet to be elucidated. Sea bass epithelial cells express MHC-IIβ, which indicates that this cell might be directly serving as an antigen-presenting cell to activate the adaptive branch of the mucosal immune system (35).

The existence of M-cells and dendritic cells in the teleost gut is controversial. Absorptive cells that resemble mammalian M-cells functionally and phenotypically have been reported in the salmonid mid intestine (21, 27, 76, 77). These fish M-like cells can uptake bovine serum albumin (27), but cannot internalize inactivated bacteria (76). Different from mammalian mature M-cells, fish M-like cells do not possess intraepithelial pockets, and thus they are thought to be morphologically similar to mammalian immature M-cells (18).

On the other hand, dendritic-like cells have been described in trout (78, 79) and zebrafish (80). These cells match mammalian dendritic cells’ hallmarks, such as activation by toll-like receptor-ligands, expression of gene signatures such as *cd83, il-1β*, *il-10r*, and *il-12 p40*, *in vivo* mobilization ability, phagocytosis ability of foreign particles, as well as the tree-like appearance (78, 81). Trout dendritic-like cells are found to express CD8α, a coreceptor of the cytotoxic T-cell (79).

Localization of antigen absorption varies among teleost species. Mucosal antigen uptake has been described in the posterior gut of European sea bass (*Dicentrarchus labrax*) (82), the second segment of the carp gut (12), the second segment of the salmonid midgut (83), the trout stomach and hindgut (84), the ballan wrasse (*Labrus bergylta*) posterior gut (85), and the Atlantic cod (*Gadus morhua*) rectum (86).

### Oral Immune Tolerance

Oral immune tolerance is a state of immunological unresponsiveness toward particular mucosal antigens, which may be due to the prevention of aberrant or excessive immune reactions to food-derived antigens or intestinal commensal microbiota, or ascribable to prior exposure to the same antigens (87). The physical-chemical barrier conferred by the GALT wards off the undesired pathogenic invasion, but also restrict the bioavailability of oral immunoprophylaxis to reach the gut inductive site for initiating local immunization. Low bioavailability of immunoprophylaxis-derived antigens is perilous as it may induce oral immunotolerance (88).

Repetitive exposure to the same antigens may induce oral tolerance. Repeated anal intubation of allogeneic cells reduced specific cytotoxicity of T-cells in carp (89, 90). Pre-exposure of fish intestinal epithelial cell line (RTgutGC) to lipopolysaccharides (LPS) strongly impeded the immunostimulatory secondary response to the LPS. Similarly, pre-treatment of a fish spleen-derived monocyte-macrophage cell line (RTS11) with LPS also lowered the transcription of cytokines. Such immune tolerance effect was not observed in both cell lines treated to the repetitive exposures to β-glucans (87).

Furthermore, prolonged exposure to orally administered antigens is another possible reason to evoke immunological tolerance (72). A study on β-glucan observed that a gradual down-regulation trend in immune gene expressions of the rainbow trout that was fed with the immunostimulant for 30 consecutive days, as compared to the gene expression levels recorded after the 15 consecutive days of post-feeding (91).

The mechanistic details of teleost oral immune tolerance remain unclear (18). It has been proposed that two major mechanisms may be involved: (1) induction of regulatory T-cells, which is often associated with repetitive exposures to the low dosage of antigen; (2) anergy or deletion, which is linked to the antigen exposure of high dose (92).

In mammalian models, mucosal T-cells have been shown to produce anti-inflammatory cytokines, particularly IL-4/13, IL-10, and TGF-β, which can induce immunosuppression and oral immune tolerance (18). These anti-inflammatory cytokines activate regulatory T-cells that eventually promote tolerance (76). The *foxp3*, a transcription factor expressed by activated regulatory T-cells, is the key marker of oral immune tolerance provoked by prolonged exposure to the same mucosal antigens. This mechanism of oral immune tolerance is termed as the induction of regulatory T-cells. In this mechanism, elevation in the *foxp3* expression level is an indicator of oral tolerance in fish (93). On the other hand, oral tolerance can be induced by T-cell anergy and apoptosis. Although this mechanism has not yet been elucidated, it has been proposed to be associated with overfeeding the antigen. The anergy or apoptosis of T-cells stimulates the production of TGF-β (92). Thus, in this mechanism, TGF-β is said to be the indicator of oral immunosuppression. The upregulation of the genes for *foxp3, il-10*, and *tgf-β* was observed in Atlantic salmon subjected to oral tolerance, inferred by the suppression of antibody production (94).

## Orally Administered Immunoprophylactics and the Implication in the Gut Immune System

### Immunostimulants

Immunoregulators are environmentally friendly compounds safe for animal utilization and can modulate the immune status of the host, and thus make the animal able to cope with diseases (95, 96). Herbal medicines have drawn much attention recently and are often administered as feed additives using a whole plant or parts of a plant (*e.g.*, leaves, fruits, seeds or root), the extracts of the plant or active compounds from the plant (97–99). Herbal compounds have been considered promising natural and effective growth promoters, antibacterial agents, and immunoprophylactic agents for finfish (100–103) and improve appetite and alleviate stress-mediated effects in fish (104). In the last few decades, investigation of the effectiveness of the application of herbal medicines as immunomodulators in aquaculture has been conducted to reduce the use of chemicals and antibiotics during production (105, 106). In fact, herbal medicine or plant extracts are rich in various biologically active substances with beneficial health properties, such as saponins, alkaloids, waxes, carotenoids, vitamin, terpenoids, tannic acid, organic acids, volatile oils, polysaccharides, glycosides, flavonoids, and others, are considered to benefit aquatic animals with improved growth and immune performance (107–110).

Many studies have reported the enhancement of systematic immunological responses in a variety of fish species after ingestion of herbal plants (or their extracts), such as increased phagocytic activity, complement activity, the ability to generate reactive oxygen and nitrogen species, lysozyme activity, antiprotease activity, and expression of immune-related genes in the blood, head kidney, spleen and liver (111–115), but only a few reported local immune parameters in the MALT. In **Table 1** we summarized studies in these past five years focusing on the immune responses of the MALT and the increased resistance to pathogenic microbes in fish that were subjected to dietary phytotherapeutics of various doses and duration times. Studies have demonstrated that dietary supplementation of medical herbs can positively influence the intestinal structure and improve the functionality of the gut. For example, Zahran et al. (129) reported that oral administration of *Withania somnifera*, commonly known as “Indian ginseng” or “winter cherry”, at a inclusion level of 5%, improved the growth of Nile tilapia, possibly due to increased levels of digestive enzymes and absorptive surface of the intestine, as well as a higher number of goblet cells (GC) in the proximal and middle gut. An elevation in the villous width in the gut from Nile tilapia (*Oreochromis niloticus*) that ingested quinoa (*Chenopodium quino*a) seeds and prickly pear fruit (*Opuntia ficus indica*) peel was reported by Ahmed et al. (118), and a higher number of GC counts in the intestine was observed in the 20% prickly pear fruit peel-supplemented group (118). Likewise, rainbow trout fed with grapevine (*Vitis vinifera*) seed extract had increased GC density and the number of intraepithelial lymphocytes in the intestine (128). GC, specialized epithelial cells play a vital role in delivering low molecular weight soluble antigens to dendritic cells in the lamina propria in the steady-state (as known as goblet-cell-associated antigen passages, GAP) in mammals (131, 132). Moreover, GCs are important in the generation of mucin which provides a thick mucus lining to the gut and protects the mucosal surface by trapping pathogenic microbes (133). A higher number of GCs was recorded in brown trout (*Salmo trutta*) intestine infected with the parasite (134) and in Nile tilapia, challenged with the bacteria *Aeromonas sobria* (118), suggesting a conserved protective role by the teleost GCs (5).

**Table 1:** Orally administrated herbs having modulatory activity on the gut immune system.

Source/form	Fish species/Body weight (g)	Doses	Duration	Results	Resistance to pathogen	Reference
*Agaricus bisporus*/powder	*Cyprinus carpio* (9.15 ± 0.09)	0-2%	8 weeks	Intestine: Expression of *gr* and *gst* ↑	N.A.	(116)
*Aloysia citrodora/*leave powder	*Oncorrhyncus myskiss* (2.5 ± 0.1)	0-2%	6 weeks	Intestine: Expression of *il-1β*, *il-8* and *tnf-α* ↑, and *tgf-β* ↓, and *il-10* –	N.A.	(117)
*Chenopodium quinoa* seed (QU) and *Opuntia ficus indica* peel (PP)/powder	*Oreochromis niloticus* (21–25)	0-20%	45 days	Intestine: Pre challenge: Intestinal villi length –, villi width ↑, goblet cell count (group PP20 only) ↑ Post challenge: Intestinal villi length and width and goblet cell count ↑	*Aeromonas sobria*	(118)
*Curcumin/*extract	*Cyprinus carpio* (16.37 ± 0.79)	0-15 g/kg	8 weeks	Intestine: Expression of *sod* and *nrf2* ↑, and CAT and *hsp70* –; Expression of *il-10* ↑, and *il-1β*, *tnf-α* and *tlr22* ↓, and *nf-κbp65* –	N.A.	(119)
*Dioscorea opposita*/powder	*Cyprinus carpio* (75.19 ± 1.56)	0-2%	8 weeks	Intestine: SOD, CAT and LYZ ↑; Total SCFA, AA, PA, BA ↑, and MDA –; MV height ↑, and muscular thickness (mid-gut) –; Expression of *oc* and *zo-1* ↑; Expression of *il-1β*, *tgf-β*, *tlr4* and *nfκb* ↑, and *il-10* and *tnf-α* – (mid-gut) Higher relative abundances of *Fusobacteria* and *Bacteroidetes*, and lower relative abundances of *Proteobacteria* (e.g. *Enterobacteriaceae*, *Shewanella*, *Pseudomonas* and *Vibrio*) and ratio of *Firmicutes*/*Bacteroidetes* in fecal microbiomes; increased the diversity of the gut flora	N.A.	(120)
*Ferula assafoetida*/powder	*Cyprinus carpio* (14.60 ± 1.29)	0-2%	8 weeks	Intestine: Expression of *lyz*, *tnf-α* and *il-1β* ↑, and *il-8* –	N.A.	(121)
*Ginkgo biloba* leaf/extract	*Epinephelus lanceolatus* ♂ *× Epinephelus fuscoguttatus* ♀ (7.84 ± 0.35)	0-10 g/kg	8 weeks	Intestine: SOD, CAT and T-AOC ↑, and MDA ↓; Expression of *zo-1*, *-2*, *-3*, *oc* and *claudin 3a* ↑ (lower conc.); Expression of *il-8* (higher conc.), *il-10* (lower conc.), *tgf-β* (lower conc.) and *tor* (lower conc.) ↑; Expression of *gpx*, *cat* and *gr* ↑ (lower conc.) and ↓ (higher conc.), but *Keap1* ↓ (lower conc.) and ↑ (higher conc.); Expression of *caspase 3*, *8* and *9* ↓ (lower conc.) and ↑ (higher conc.)	N.A.	(122)
*Ginkgo biloba* leaf/extract	*Cyprinus carpio* (7.84 ± 0.35)	0-10 g/kg	8 weeks	Intestine: Expression of *il-1β*, *il-8*, *tnf-α*, *il-10*, *tgf-β*, *inos*, *cox2* and *arg* ↓, and *saa*, *hep*, and *gpx1* ↑, and *sod* –; SUR ↑	*Aeromonas hydrophila*	(123)
*Jasonia glutinosa/*powder	*Sparus aurata L.* (6.0 ± 0.8)	0-30%	15 and 30 days	Intestine: Expression of *cat* and *sod* ↑ in the PrI on day 15	N.A.	(124)
*Prunus domestica*/extract	*Oncorhynchus mykiss* (27.61 ± 0.44)	0-1%	21 days	Intestine: Expression of *il-10*, *il-6*, *il-8*, *il-12* and *cox-2* ↑, and *il-1β* –; SUR ↑	*Yersinia ruckeri*	(125)
*Psidium guajava/*leaf extract	*Oreochromis niloticus* (1.32 ± 0.04)	0-1%	84 days	Intestine: Insignificantly increased villi height and width; SUR ↑	*Aeromonas hydrophila*	(126)
*Psidium guajava/*leaf powder	*Cyprinus carpio* (15.88 ± 0.27)	0-1%	8 weeks	Intestine: Expression of *il-1β* and *il-8* ↑, and *tnf-α* –	N.A.	(116)
*Silybum marianum* L./extract	*Ctenopharyngodon idella* (24.2 ± 0.1)	0-100 mg/kg	70 days	Intestine: InL, ILI, IW and ISI↑ Mucosal permeability ↓ Improved the intestinal histological pathological symptoms after infection; Expression of 9 TJ-related genes (*zo-1*, *-2b*, *oc*, *jam-a*, *claudin-b*, *-c*, *-f*, *-3c*, *-11*) ↑, and 2 (*claudin-12* and *-15a*) ↓, and *claudin-7a*, *-7b* and *-15b* –; Expression of 5 AJC-related genes (*e-cadherin*, *α-catenin*, *β-catenin*, *nectin*, *afadin*) ↑; Expression of 4 AJC-related genes (*rhoa*, *rock*, *mlck* and *nm-ii*) ↓; GTP-RhoA protein levels ↓	N. A.	(127)
*Vitis vinifera* seed/extract	*Oncorhynchus mykiss* (~ 1.3)	0-200 mg/kg	60 days	Intestine: Villus height (PrI, MI, DI) and width (PrI, DI) ↑, Tunica muscularis thickness, absorption surface area, villus density, acidic/neutral/mixed mucin goblet cells – Goblet cell density (PrI, DI) ↑, Number intraepithelial lymphocytes (PrI) ↑; Expression of *c3*, *lyz* and *ifn-γ* –, and *β-defensin3*, *tnf-α* ↑	N. A.	(128)
*Withania somnifera* root*/*powder	*Oreochromis niloticus* (45)	0-5%	60 days	Intestine: Diameter of lumen ↓ in the DI, and – in the PrI and MI; Number of mucosal folds: ↑ in the PrI, but ↓ in the MI and DI; Number of goblet cells: ↑ in the PrI and MI, but – in the DI; Mucosal folds height: – in the PrI, but ↑ in the MI and DI; Perimeter: – in the PrI and MI, but ↑ in the DI; Area within the perimeter for each fold: – in the PrI and MI, but ↑ in the DI; Width of lamina propria: ↑ in the PrI, MI and DI; Thickness of muscle: ↓ in the PrI, but –MI and DI	*Streptococcus iniae* (only study the expression of cytokines in the head kidney and spleen)	(129)
*Yucca schidigera/*extract	*Cyprinus carpio* (45.21 ± 0.43)	0-400 mg/kg	8 weeks	Intestine: T-AOC, C3, C4 and LYZ ↑, and MDA ↓, and total SOD and IgM –; Expression of *zo-1*, *oc*, *claudin 11* ↑ (TJ genes), *claudin -3* and -*7* –; Expression of *tgf-β2* ↑, *il-10* –, and *il-6*, *il-1β* and *tnf-α* ↓ (inflammatory cytokine genes); Expression of *CuZnsod*, *cat*, *gpx1a* and *nrf2* ↑, and *keap1* ↓ (antioxidant genes);	N.A.	(130)

Few studies have evaluated the influence of dietary supplementation of medicinal plants on the expression of junctional genes in the intestine. Meng et al. (120) investigated the effects of oral administration of yam (*Dioscorea opposita*) peels to common carp (*Cyprinus carpio*) and found higher microvilli density and GC numbers as well as elevated transcript levels of tight junction (TJ)-related genes (*occludin* (*oc*) and *zonula occludens-1* (*zo-1*) (118). Similar findings were also reported in common carp fed *Yucca schidigera* (known as the Mojave yucca or Spanish dagger) extract in the feed, with higher gene expression levels of *zo-1*, *oc*, and *claudin 11* in the intestine after an 8-week feeding trial (130). In hybrid grouper (*Epinephelus lanceolatus* ♂ *× E. fuscoguttatus* ♀), *zo-1*, *-2*, *-3*, *oc* and *claudin 3a* were induced in the gut when fed with high lipid diets supplemented with *Ginkgo biloba* (maidenhair tree) leaf extract (122). Dietary supplementation with silymarin (extracted from *Silybum marianum* L.) improved the growth of juvenile grass carp possibly owing to promoted intestinal histology (127). Moreover, ingestion of silymarin induced transcript levels of barrier-forming tight junction (TJ)- and adherent junction (AJ)- related genes accompanied by the reduced expression of pore-forming TJ genes by inhibiting a small Rho GTPase protein (RhoA) and/Rho-associated protein kinase (ROCK) signaling pathway in the gut, indicating that silymarin treatment could enhance intestinal apical junctional complex (AJC) integrity by strengthening TJ and AJ. (127). These findings indicate that supplementation of certain herbal medicines can improve the growth of fish and equally important, strengthen the immune status of the gut.

Cytokines can be categorized into proinflammatory cytokines (*e.g.*, IL-1β, IL-8, TNF-α, and IL-6) and anti-inflammatory cytokines (*e.g.*, IL-10 and TGF-β) (130, 135). Cytokines are crucial for regulating multiple aspects of the immune response. Thus, they have been monitored to predict changes in the gut immunity. Studies have shown the anti-inflammatory effects in the gut after the use of herbal medicinal products. TGF-β1 and IL-10 are immunosuppressive cytokines that restrain inflammation by decreasing the production of inflammatory cytokines (135). Significantly downregulated mRNA expression of proinflammatory cytokines (*e.g.*, *il-1β* and *tnf-α*) and upregulation of anti-inflammatory cytokines (*e.g.*, *il-10* and *tgf-β*) were observed in the intestine of common carp after 8 weeks of curcumin administration (119), *Yucca schidigera* extract (130) and *Ginkgo biloba* leaf extract (123). Similarly, hybrid groupers fed *Ginkgo biloba* leaf extract also exhibited higher expression of *il-10* and *tgf-β* in the intestine (122).

However, the opposite expression pattern of cytokines in herbal medicine treated fish has also been reported. Increased transcript levels of proinflammatory cytokines were reported in the intestine of common carp with *Psidium guajava* (guava) leaf powder (116), *Dioscorea opposita* (chinese yam) powder (120) or *Ferula assafoetida* (asafetida) powder (121), and rainbow trout with *Aloysia citrodora* (lemon verbena) leave powder (117) or *Vitis vinifera* seed extract (128). IL-1β is an effector in the inflammatory responses expressed by distinct cell populations after the activation of pattern recognition receptors (PRRs) once triggered by an invading pathogen. IL-6 is known to play a major role in haematopoiesis e.g. promoted macrophage growth (136), and is with biphasic pro-and anti-inflammatory properties (135). IL-8 is a chemokine for attracting neutrophils, monocytes, basophils, T cells, and eosinophils (135). TNF-α, with overlapping functions with IL-1β, is an immune gene expressed in the early phase of infection and has a key role in regulating inflammation (135). However, TNF-α can activate NADPH oxidase, which leads to the generation of reactive oxygen species (ROS) that may promote inflammation by activating inflammasomes and the release of mature IL-1β and IL-18 cytokines (137–139), as well as serving as second messengers to control the action of several signaling pathways (140). Therefore, an increased expression level of inflammatory cytokines in the intestine can have negative health effects for fish (141).

### Vaccines and Adjuvants

Vaccination is one of the essential and powerful prophylactic means of infectious disease control that can provoke immune memory and reduce the need for antibiotics in aquaculture (142). Many types of vaccines have been developed with different ways of introduction, including spray, oral, immersion and injection. Although injectable vaccines have been proven to be effective, some critical limitations such as the requirement of high labor demand, trauma on the skin at the injection site that may cause secondary infection, fish size at vaccination and handling stress to the fish, have also been noticed (143). Oral immunization can circumvent the aforementioned disadvantages; therefore, is now an important topic under investigation (13). However, there are still some obstacles that require solutions to achieve high efficacy of oral vaccines (77).

The antigens in a vaccine may be broken down and inactivated in the gastrointestinal tract before reaching the intestinal mucosa and activating immune cells (144). The availability of antigens will thus be lowered, and a low antigen dose will induce regulatory T-cell-mediated immune tolerance (92). To protect the antigens from gastric degradation, several encapsulation techniques such as using alginate microparticles, virus-like particles (VLPs), chitosan, liposomes, immunostimulating complexes (ISCOMs) and poly (D, L-lactic-co-glycolic acid) (PLGA) have been developed and summarized previously (92, 145, 146). ISCOMs are formulated by the mixing of; amphipathic antigen, the saponin-based adjuvant Quil-A, and cholesterol in a 1:1:1 ratio (147). ISCOM technology-based Matrix M™ adjuvant has been studied in a range of veterinary vaccines and has the potential to be commercialized (147).

In terms of antigen production, subunit antigens are particularly of interest since they can be produced using various protein expression systems and are safe to the host given the fact that they do not possess live components of the pathogen (148). *Escherichia coli* (*E. coli*) (149) and yeast (150, 151) are two of the most widely used protein expression systems for the production of subunit antigens in fish vaccinology (92), but recent studies have also shown the potential of using other enteric probiotics as vaccine vehicles.

Choosing the right enteric probiotic as a vaccine vehicle is important as fish gastrointestinal mucus dissociates microbes and is constantly sloughed off and replaced. The transient availability or low doses of vaccine immunogens in the inductive site of the gastrointestinal tract will result in poor antigen uptake by the GALT and thus diminish the success rate of immunization. Generally regarded as safe (GRAS) is a United States Food & Drug Administration (FDA) designation stipulates that any substance that is intentionally added to food is generally considered safe by qualified experts. Lactic acid bacteria (LAB), e.g. *Lactococcus* and *Lactobacillus* (152) and *Bacillus subtilis* (153) are recognized as probiotics, which are GRAS to fish and thus can be used as an oral vaccine vehicle to present antigen (**Table 2**). LAB not only can colonize and persist in the gastrointestinal tract, but with prospective applications such as modulating the host immune system, competing with pathogens for mucosal binding sites, promoting digestive function, improving the disease resistance of the host, delivering expression DNA and antigen presentation to mucosal tissue of the host (152, 154, 170, 171).

**Table 2:** Oral vaccines and the feeding regimes.

Source/form	Fish species/Body weight (g)	Doses	Feeding regime	Results	Resistance to pathogen	Reference
*Lactococcus lactis* BFE920 expressing *Edwardsiella tarda* OmpA, FlgD, or a fusion antigen of the two	*Paralichthys olivaceus* (86.36 ± 4.31)	~0.8-1.4 × 10^7^ CFU/g	Twice with a one-week interval	Intestine: Expression of T cell responses (*cd4-1*, *cd4-2*, *cd8-α*, *t-bet*, *ifn-γ*) ↑ Expression of *tlr5m*, *il-1β* and *il-12p40* ↑ SUR ↑ after being challenged at 4^th^ week	*Edwardsiella tarda*	(142)
*Lactococcus lactis* expressing the G gene from VHSV	*Oncorhynchus mykiss* (7 ± 0.65)	1.0 × 10^10^ CFU/g and 1.0 × 10^8^ CFU/g	Vaccination was conducted on day 1–7 and day 15–21	SUR ↑ after being challenged on day 60 post vaccination	VHSV	(154)
*Lactococcus lactis* expressing HIRRV-glycoprotein (G) on the cell surface	*Paralichthys olivaceus* (35 ± 5)	1.0 × 10^9^ CFU/g diet	Vaccination was conducted at week 1 and week 5	Intestine: IgM against HIRRV ↑ in the gut mucus SUR ↑ after being challenged at 8^th^ week	HIRRV	(155)
*Lactococcus lactis* BFE920 expressing OmpK and FlaB	*Paralichthys olivaceus* Juvenile (7.1 ± 0.8 g) and adult (140 ± 10 g)	1 × 10^7^ CFU/g feed	Vaccination was conducted for 1 week with a 1-week interval, repeating three times	Intestine: Expression of T cell responses (*cd4-1*, *cd4-2*, *cd8-α*, *t-bet*, *ifn-γ*) ↑ Expression of *tlr5m*, *il-1β* and *il-12p40* ↑ SUR ↑ after being challenged at 7^th^ week	*Vibrio anguillarum*, *Vibrio alginolyticus*, and *Vibrio harveyi*	(156)
*Lactobacillus casei* expressing the fusion protein of OmpI from *A. veronii* TH0426 and C5 – I from common carp (surface-displayed or secreted)	*Carassius carassius* (50.00 ± 1.00 g)	~1.0 × 10^9^ CFU/g feed	Vaccination was conducted on days 1–3 (prime vaccination), 18–20 (booster vaccination) and 34 (challenge)	Intestine: Expression of *il-1β*, *il-10*, *ifn-γ* and *tnf-α* ↑post vaccination SUR ↑ after being challenged on day 34	*Aeromonas veronii*	(157)
*Lactobacillus casei* strain expressing Malt from *Aeromonas veronii* TH0426	*Cyprinus carpio* (65 ± 4)	~1.0 × 10^9^ CFU/g feed	Vaccination was conducted on days 0–2 (prime vaccination), 14–15 (booster vaccination) and 34 (challenge)	Intestine: Expression of *il-1β*, *il-10*, *ifn-γ* and *tnf-α* ↑post vaccination SUR ↑ after being challenged on day 34	*Aeromonas veronii*	(158)
*Lactobacillus casei* expressing the OmpW of *A. veronii* (surface-displayed or secreted)	*Cyprinus carpio* (56 ± 1)	1.0 × 10^9^ CFU/g feed	Vaccination was conducted on day 1–3 and on day 18–20	Intestine: Expression of the *il-1β*, *il-10*, *ifn-γ* and *tnf-α* ↑ post vaccination SUR ↑ after being challenged on day 34 after the boost immunization	*Aeromonas veronii*	(159)
*Lactobacillus casei* expressing the FlaB of *A. veronii* (surface-displayed or secretory)	*Cyprinus carpio* (56 ± 1)	2.0 × 10^9^ CFU/g feed	Vaccination was conducted on day 0–2 and on day 28–29	Intestine: Expression of the *il-1β*, *il-10*, *ifn-γ* and *tnf-α* ↑ post vaccination SUR ↑ after being challenged on day 58 after immunization	*Aeromonas veronii*	(160)
*Lactobacillus casei* expressing the OmpAI of *A. veronii* (surface-displayed or secretory)	*Cyprinus carpio* (50 ± 1)	2.0 × 10^9^ CFU/g feed	Vaccination was conducted on day 1 and on day 32	Intestine: Expression of the *il-1β*, *il-10*, *ifn-γ* and *tnf-α* ↑ post vaccination SUR ↑ after being challenged on day 66 after immunization	*Aeromonas veronii*	(161)
*Lactobacillus casei* expressing CK6-VP2 fusion protein	*Oncorhynchus mykiss* (11.5)	2 × 10^9^ CFU/fish	Vaccination was conducted on days 1 and 32	Intestine: Expression of *β-defensin* ↑ Skin mucus: Titer of anti-VP2 IgT ↑	IPNV	(162)
*Lactobacillus casei* expresses the AHA1-CK6-VP2 fusion protein	*Oncorhynchus mykiss* (~ 10)	200 μl of recombinant strains	Orogastric intubation was conducted on days 1, 2 and 3, and boost on days 31, 32, and 33	Intestine: The colonization ability of pPG-612-AHA1-CK6-VP2/*L. casei* 393 was higher than other groups on day 3 and 7 Skin mucus: Titer of anti-VP2 IgT ↑	IPNV	(144)
*Lactobacillus rhamnosus* expresses the ORF81 from CyHV3	*Cyprinus carpio* (~ 50)	5.2 10^10^ CFU/g feed	Vaccination was conducted on day 1-3, day 14-16 (booster vaccination) and day 28-30 (booster vaccination)	SUR ↑ after being challenged on day 15 after the second booster	CyHV3	(163)
*Bacillus subtilis* spores displaying the VP7 of GCRV	*Ctenopharyngodon idella* (50 ± 5)	1.0 × 10^10^ spores/fish	Vaccination was conducted on day 1 and day 8	SUR ↑ after being challenged on day 14 after the boost immunization	GCRV	(164)
*Bacillus subtilis* spore expressing the VP4 of GCRV	*Ctenopharyngodon idellus* (23 ± 2)	2.3 × 10^11^ spores/fish/day, amount to 1 × 10^−3^ μg/g (protein/fish)	Vaccination was conducted daily for 8 weeks	Intestine: Lower GCRV viral load in intestine Higher anti-GCRV IgZ titer in the intestinal mucus Expression of *csf1r*, *mhc-ii*, *cd8* and *il-1β* ↑ SUR ↑ after being challenged on week 10	GCRV	(165)
*Bacillus subtilis* GC5 expressing the Sip of *S. agalactiae* on the surface	*Oreochromis niloticus* (22 ± 2)	10^9^ CFU/100 μL/fish	Vaccination was conducted once on week 0 and once on week 3	Intestine: Expression of *tp3*, *tnf-α*, *il-1β*, *tgf-β*, *il-10*, *mhc-i*, *mhc-ii*, *cd4*, *cd8*, *tcr-β*, *igm* and *t-bet* were differentially modulated post vaccination SUR ↑ after being challenged on week 6 post-vaccination	*Streptococcus agalactiae*	(166)
Yeast expressing the OmpG and Omp48 of *A. hydrophila*	*Carassius auratus* (~6)	1.5 × 10^8^ heat-killed yeast cells/g meal powder	Vaccination was conducted daily for 4 weeks	SUR ↑ after being challenged on day 28	*Aeromonas hydrophila*	(150)
*Saccharomyces cerevisiae* expressing ORF131 of CyHV-3 on the cell surface	*Cyprinus carpio* var. Jian (~10)	1.6 × 10^9^ CFU yeast in 300 μL PBS	Vaccination was conducted 3 times at a 2-week interval	SUR ↑ after being challenged at four weeks post the third immunization	CyHV-3	(151)
Rootless duckweed (Wolffia) expresses LamB from *Vibrio alginolyticus*	*Danio rerio* (N.A.)	N. A.	Vaccination was conducted for 60 days from month 0-2 and boost for 30 days on month 3-4	SUR ↑ after being challenged six weeks post-vaccination (booster)	*Vibrio alginolyticus*	(167)
Tobacco leaves express RGNNV- capsid protein	*Epinephelus septemfasciatus* (25.8 g)	200 μg/fish	Vaccination was conducted once a day for five consecutive days	SUR ↑ after being challenged on day 21 after immunization	RGNNV	(168)
*Escherichia coli* expressing the capsid protein of NNV	*Dicentrarchus labrax* (10–12)	10^10^ CFU/g diet	Vaccination was conducted 3 consecutive days and a boost at day 14	SUR ↑ after being challenged at 30 days post vaccination	NNV	(149)
Tobacco expressing the capsid protein of NNV	*Hyporthodus septemfasciatus* (N.A.)	5 µg or 10 µg plant-derived recombinant coat protein	Vaccination was conducted every Monday at 2-week intervals for a total of 4 times	SUR ↑ after being challenged at six days after the final immunization	NNV	(169)

*Lactococcus lactis* is a widely used bacterium and is a prominent candidate to develop oral vaccines and host-vector, since it possesses several advantages, such as: absence of endotoxins and biogenic amine production, and ability to be cultured in chemically defined media, it can be genetically manipulated, the genome sequence is readily available and is considered to have a safe profile for use in the development of vaccine formulations (154, 172–174). Successful examples of immunization and induction of protection of fish using *Lactococcus lactis-*expressing antigen(s) from *Edwardsiella tarda* (outer membrane protein (Omp)A and flagellar hook protein (Flg)D) (142), viral hemorrhagic septicemia virus (VHSV) (154), and hirame novirhabdovirus (HIRRV) (155) have been reported. These studies demonstrated that the immunization elicited higher expression of T cell markers and proinflammatory genes in the intestine (142) and higher survival rate relative to the control group in the challenge assay a few weeks post-vaccination. Interestingly, a recent study showed that oral immunization of olive flounder (*Paralichthys olivaceus*) with *Lactococcus lactis* BFE920 that express fusion antigens of OmpK from *Vibrio anguillarum* and flagellin B subunit (FlaB) from *Vibrio alginolyticus* increased the levels of serum antigen-specific antibodies and expression of cytokines and T cell markers in the intestine. It is important to highlight that the same study revealed the universal protective effects of the vaccine to fish from multiple strains of *Vibrio* pathogens, namely *Vibrio anguillarum*, *Vibrio alginolyticus*, and *Vibrio harveyi*, even though the vaccine did not contain specific antigens from *Vibrio harveyi*. The cross-protection against *Vibrio harveyi* may happen due to the high homologies in protein sequences and structures of the OmpK and FlaB among the three *Vibrio* species, which then rendered in immunological cross-reactivity *via* shared epitopes (156). Therefore, comprehensive antigen mapping is encouraged for developing universal vaccines in fish with high protection.

The literature accumulates evidence that *Lactobacillus casei* is also a prominent live vehicle for expressing and transporting heterologous antigens to mucosal sites. For example, the recombinant *Lactobacillus casei* can be detected in the digestive tract of common carp following oral administration, and colonization was shown to be higher in the hind-gut than in the prosogaster and mid-gut (158–161). Meanwhile, these studies also showed that oral vaccination of common carp with *Lactobacillus casei* (1-2 × 10^9^ CFU/g feed) displaying antigen(s) (surface-displayed or secreted) from *Aeromonas veronii* resulted in elevated transcript level of immune genes in the intestine post-vaccination and provided strong protection for common carps against this pathogenic bacteria (158–161). Immunization of crucian carp (*Carassius carassius*) with recombinant *Lactobacillus casei* that expresses a fusion protein encoded by of *OmpAI* gene from *Aeromonas veronii* and chemokine *c5 – i* gene (served as a molecular adjuvant) from common carp resulted in enhanced alkaline phosphatase, superoxide dismutase and acid phosphatase in the serum and higher expression of *il-10*, *il-1β*, *tnf-α*, and *ifn-γ* in the heart, liver, spleen, head kidney, and intestinal tract. More importantly, recombinant *Lactobacillus casei* provided strong protection (survival rate at least 60% versus 0% for the unimmunized control group) against *A. veronii* infection (157).

Additionally, *Lactobacillus casei* was proven to be able to express a fusion protein (VP2-CK6) of a viral gene (VP2) from infectious pancreatic necrosis virus (IPNV) and a chemokine (CK6) gene from rainbow trout that can induce leukocyte migration, inflammatory responses and killing the target cells (162). Orally vaccinated fish exhibited induced expression of *β-defensin* in the intestine, higher anti-VP2 IgT titer in the skin mucus and lower viral load in the liver and pancreas compared to the control group (162). To further boost the effects of this oral vaccine, a genetically engineered *Lactobacillus casei* was constructed to present the *Aeromonas hydrophila* adhesion (AHA1) *-*CK6*-*VP2 fusion protein (144). Recombinant AHA1 protein was illustrated to adhere to epithelial cells possibly due to its strong hydrophobicity that can bind to the cell surface receptor *via* covalent bonds (175). Intestinal colonization and the ability to induce specific anti-IPNV-specific IgT and IgM antibodies were found to be higher in the fish that had AHA1-CK6-VP2 expressed *Lactobacillus casei* than other groups (including the *Lactobacillus casei* that displayed VP2-CK6 recombinant protein), indicating that AHA1 helped in antigen retention in the intestinal tract and enhance the immunogenicity of the LAB vaccine (144).

The utilization of probiotic vaccines provided a certain tolerance to harsh conditions in the gastrointestinal tract. However, better cell viability of probiotics during passing through the gastrointestinal tract would improve their efficacy. For example, oral immunization of Koi carp (*Cyprinus carpio*) with chitosan-alginate encapsulated live recombinant *Lactobacillus rhamnosus* expressing ORF81 protein from cyprinid herpesvirus 3 (CyHV-3) provided elevated antigen-specific IgM production in the serum and antigen-specific IgM-secreting cells in the spleen (163). A higher survival rate was noted for the fish orally vaccinated with the encapsulated live probiotic vaccine than that of fish fed with the vector-containing probiotic control group after CyHV-3 challenge (163). However, it is worth mentioning that a comparison of recombinant probiotic vaccines containing specific antigens (ORF81 in this case) with or without encapsulation would provide valuable information on the efficacy of encapsulation. The spores of *Bacillus subtilis* can withstand wet heat, desiccation and tolerate acid conditions. Additionally, *B. subtilis* spores exhibit the potent adjuvant property that can benefit by inducing the protective immune responses and minimizing tolerance (176), which makes it an ideal antigen producing and delivering system for the fish oral vaccine (165, 166). Research has demonstrated that oral vaccination of grass carp (*Ctenopharyngodon idella*) with the engineered *B. subtilis* spores that express VP7 (164) or VP4 (165) from grass carp reovirus (GCRV) on the spore surface could provide adequate immunity against GCRV infection; although, the challenge assay, in these two studies, was assessed for a very short time (14 days) following the last vaccine administration. These works highlighted a novel strategy of applying LAB and *Bacillus subtilis* spores, two powerful and efficient expression systems as oral vaccine delivery vehicles, which confer high immunogenicity and sufficient protection against microbial infection.

An adjuvant is purported to be crucial in improving immunogenicity and prolonging the duration of protection of a mucosal vaccine (177). Choosing the right combination of adjuvant and vaccine candidates will balance and circumvent oral tolerance (145). Aluminum hydroxide and oil-based adjuvants are the most commonly used adjuvants in injectable vaccination of aquaculture due to their high efficacy and low production cost (178). These adjuvants are less commonly used in oral vaccine studies as they can result in local negative effects such as necrosis and tissue inflammation (145). Recombinant cytokines are gaining popularity as the ideal mucosal adjuvants as they do not induce necrosis. Although synthetic cytokines have been long applied as an adjuvant in the injectable vaccine in aquaculture, the first fish study that evaluated the recombinant cytokine as an oral adjuvant was reported by Galindo-Villegas et al. (179). By incorporating recombinant TNF-a with a commercial oral vaccine of *V. anguillarum*, higher immunostimulatory responses including *il-1β*, *lysozyme* and *IgT* production were recorded in the adjuvant group relative to the non-adjuvanted group (179). Other pro-inflammatory cytokines such as IL-12 and IFN-γ have been proposed to be potential oral vaccine adjuvants (145).

Plants have been applied as bioreactors to produce biopharmaceuticals including antigens for vaccines, growth factors, antibodies, and cytokines (180). With thick and rigid cell walls, transgenic plants are regarded as one of the ideal solutions for antigen generation and protection simultaneously (167). Additional advantages such as cost-effectiveness, high scalability, and low risk of contamination by bacterial components (e.g. endotoxins) are also proposed for plant molecular farming (180). Feeding zebrafish with rootless duckweed (*Wolffia globosa*) expressing LamB (maltoporin) from *Vibrio alginolyticus* resulted in high relative percent survival (RPS) of the vaccinated fish (63.3%) from *Vibrio* infection (167). Similarly, oral administration of the crudely purified protein extract containing chloroplast-derived red-spotted grouper NNV (RGNNV) virus-like particle (VLP) provided comparable protection compared to a commercial injectable vaccine in the sevenband grouper fish against RGNNV challenge (168).

## Challenges and Future Perspectives

Despite the growing number of oral prophylactics being reported, the lack of consistency in performance, particularly on-site farm testing, and the limited successful application remain pressing issues (181). As the most diverse and largest vertebrate groups (182), fish display high heterogeneity in their physiology and immune system (38). For instance, the gadoid species do not possess CD4 (53) and MHC molecules (53, 183) compared to the other finfish species. Anatomically, cultured finfish can be differentiated as gastric and agastric species, which differ significantly in the morphology and structure of their gastrointestinal tract (38). As mentioned earlier, the nomenclature applied for dividing the intestine in fish has been inconsistent. These aforementioned factors have driven the divergence in the findings of fish gut immunity. The functionality and underlying mechanistic details of some GALT immune components, viz. teleost IgD, remains obscure.

Immunological studies of fish are slower than those of their mammalian counterparts. Furthermore, insights derived from mammalian immune studies may not be applicable to aquaculture. CD8-α^+^, a signature receptor of mammalian cytotoxic T-cells have been reported to be expressed by fish dendritic cells beside cytotoxic T-cells (79). The lack of specific cellular biomarkers to differentiate leukocyte subpopulations in many aquaculture species impedes the understanding of the gut immune system in higher resolution (184).

Even though teleost fish were the first vertebrate animals to start presenting the classic adaptive immunological features, their antibody isotypes are more limited and primitive compared to other animals on the upper scale of the evolutionary tree (185); Thus, their adaptive immunological responses are not as effective as other farmed livestock species like poultry, swine or cattle, and the immunological memory might not be long-lasting. In addition, fish gut possesses intricate GALT and a harsh gastrointestinal environment to fend off the microbial intrusion, but it also greatly reduces the uptake of the immunoprophylactics at the induction site (38). To provoke the desired bioactivity and prevent oral tolerance, the research and development of oral immunoprophylactics must address several necessities, such as a substantial amount of antigen, proper encapsulation or vector and a well-designed feeding regime (38). To elicit long-lasting protection, there are trade-offs between repetitive immunizations *via* booster and the risk of getting anergy-mediated immune suppression. In addition to these challenges, the design and technology of the orally administered end prophylactics should be cost-effective to prevent overtaxing aquaculture production costs.

Aquaculture practices worldwide are generating a diverse range of finfish species. These species of finfish evolved differently, in which the developmental biology of these cultured finfish can have particularities. The differences in phenotype, biological processes and responses, and molecular functions are associated with the control and regulation at the molecular level, where the epigenome can play role. Thus, research findings from the study of one finfish species do not always translate to another species. Therefore, extensive study of various aspects, which include the fundamental immunology and functional characterization; physiology; biomarker development; and the optimization of the feeding regime as well as rearing conditions are necessary for each species of interest.

## Author Contributions

P-TL shares the first authorship. FY and C-FL have contributed equally to this work. J-YL and C-MC share the last and corresponding authorship. All authors contributed to the article and approved the submitted version.

## Conflict of Interest

The authors declare that the research was conducted in the absence of any commercial or financial relationships that could be construed as a potential conflict of interest.

## Publisher’s Note

All claims expressed in this article are solely those of the authors and do not necessarily represent those of their affiliated organizations, or those of the publisher, the editors and the reviewers. Any product that may be evaluated in this article, or claim that may be made by its manufacturer, is not guaranteed or endorsed by the publisher.

## Glossary

**Table:**

AA	acetic acid
AHA1	*Aeromonas hydrophila* adhesion
AJC	apical junctional complex
ARG	arginase
BA	butyric acid
C3	Complement 3
C4	Complement 4
CAT	catalase
CD	cluster of differentiation
CFU	colony forming unit
CK6	Chemokine 6
COX	cyclooxygenase
CSF1R	colony-stimulating factor 1 receptor
CuZnSOD	copper zinc superoxide dismutase
CyHV-3	Cyprinid Herpesvirus 3
DI	distal intestine
flaB	flagellin B
FlgD	flagellar hook protein D
GALT	gut-associted lymphoid tissues
GCRV	grass carp reovirus
GPx	glutathione peroxidase
GR	glutathione reductase
GST	glutathione S-transferases
HEP	hepcidin
HIRRV	Hirame novirhabdovirus
HSP	heat shock protein
IEL	intraepithelial lymphocyte
IFN	interferon
Ig	immunoglobulin
IL	interleukin
ILI	intestinal length index
InL	intestinal length
iNOS	inducible nitric oxide synthase
IPNV	Infectious pancreatic necrosis virus
ISI	intestinal somatic index
IW	intestinal weight
JAM-A	junctional adhesion molecule-A
Keap1	Kelch-like- ECH-associated protein 1
Lamb	Lambda B
LPS	lipopolysaccharides
LYZ	lysozyme
MDA	malondialdehyde
MHC	major histocompatibility complex
MI	middle intestine
MLCK	myosin light chain kinase
MV	microvillus
N.A.	Not available
NFκB	nuclear factor kappa B
NMII	non-muscle myosin II
NNV	Nerve necrosis virus
Nrf2	nuclear factor erythroid 2–related factor 2
OC	occluding
Omp	outer membrane protein
ORF131	open reading frame 131
PA	propionic acid
PBS	phosphate buffered saline
PP20	prickly pear fruit peel-supplemented group (20%)
PrI	proximal intestine
RGNNV	red-spotted grouper nervous necrosis virus
RhoA	a small Rho GTPase protein
ROCK	the Rho associated protein kinase
SAA	serum amyloid A
SCFA	short chain fatty acids
Sip	surface immunogenic protein
SOD	superoxide dismutase
SUR	survival rate
T-AOC	total antioxidant capacity
T-bet	T-box expressed in T cells
TCR-β	T-cell receptor β chain
TGF	transforming growth factor
TJ	tight junction
TLR	toll-like receptor
TLR5M	membrane form of TLR5
TNF	tumor necrosis factor
TOR	target of rapamycin
TP3	Tilapia piscidin 3
TRγδ	T-cell receptors of the γδ heterodimers
VHSV	viral haemorrhagic septicaemia virus
VP2	viral protein 2
ZO	zonula occludens

## References

[1] Lafferty KD Harvell CD Conrad JM Friedman CS Kent ML Kuris AM. Infectious Diseases Affect Marine Fisheries and Aquaculture Economics. Ann Rev Mar Sci (2015) 7:471–96. doi: 10.1146/annurev-marine-010814-015646 25251276

[2] Behringer DC Wood CL Krkošek M Bushek D. Disease in Fisheries and Aquaculture. In: Marine Disease Ecology. Oxford: Oxford University Press (2020). p. 1–183.

[3] Foey A Picchietti S. Immune Defences of Teleost Fish. In: Merrifield DL Ringø E, editors. Aquaculture Nutrition: Gut Health, Probiotics and Prebiotics. Hoboken: Wiley Online Library (2014). p. 14–52.

[4] Bøgwald J Dalmo RA. Gastrointestinal Pathogenesis in Aquatic Animals. In: Merrifield DL Ringø E, editors. Aquaculture Nutrition: Gut Health, Probiotics and Prebiotics. Hoboken: Wiley-Blackwell Publishing Oxford, UK (2014). p. 53–74.

[5] Salinas I Parra D. Fish Mucosal Immunity: Intestine. In: Beck BH Peatman E, editors. Mucosal Health in Aquaculture. San Diego: Academic Press (2015). p. 135–70.

[6] Krogdahl Å Penn M Thorsen J Refstie S Bakke AM. Important Antinutrients in Plant Feedstuffs for Aquaculture: An Update on Recent Findings Regarding Responses in Salmonids. Aquac Res (2010) 41(3):333–44. doi: 10.1111/j.1365-2109.2009.02426.x

[7] Trushenski J. Nutritional Impacts on Fish Mucosa: Dietary Considerations. In: Beck BH P E, editors. Mucosal Health in Aquaculture. London: Elsevier (2015). p. 199–209.

[8] Humphries F Benzie JA Morrison C. A Systematic Quantitative Literature Review of Aquaculture Genetic Resource Access and Benefit Sharing. Rev Aquac (2019) 11(4):1133–47. doi: 10.1111/raq.12283

[9] Picchietti S Miccoli A Fausto AM. Gut Immunity in European Sea Bass (*Dicentrarchus Labrax*): A Review. Fish Shellfish Immunol (2020) 108:94–108. doi: 10.1016/j.fsi.2020.12.001 33285171

[10] Cain K Swan C. Barrier Function and Immunology. In: Grosell M Farrel AP Brauner C, editors. Fish Physiology, vol. 30. London: Academic Press (2010). p. 111–34.

[11] Díaz AO Garcia AM Devincenti CV Goldemberg AL. Morphological and Histochemical Characterization of the Mucosa of the Digestive Tract in *Engraulis Anchoita* (Hubbs and Marin). Anat Histol Embryol (2003) 32(6):341–6. doi: 10.1111/j.1439-0264.2003.00490.x 14651481

[12] Rombout JH Abelli L Picchietti S Scapigliati G Kiron V. Teleost Intestinal Immunology. Fish Shellfish Immunol (2011) 31(5):616–26. doi: 10.1016/j.fsi.2010.09.001 20832474

[13] Yu Y Wang Q Huang Z Ding L Xu Z. Immunoglobulins, Mucosal Immunity and Vaccination in Teleost Fish. Front Immunol (2020) 11:2597. doi: 10.3389/fimmu.2020.567941 PMC7566178 33123139

[14] López Nadal A Ikeda-Ohtsubo W Sipkema D Peggs D McGurk C Forlenza M. Feed, Microbiota, and Gut Immunity: Using the Zebrafish Model to Understand Fish Health. Front Immunol (2020) 11:114. doi: 10.3389/fimmu.2020.00114 32117265 PMC7014991

[15] Bereded NK Curto M Domig KJ Abebe GB Fanta SW Waidbacher H. Metabarcoding Analyses of Gut Microbiota of Nile Tilapia (*Oreochromis Niloticus*) From Lake Awassa and Lake Chamo, Ethiopia. Microorganisms (2020) 8(7):1040. doi: 10.3390/microorganisms8071040 PMC7409238 32668725

[16] Bowyer PH El-Haroun ER Salim HS Davies SJ. Benefits of a Commercial Solid-State Fermentation (SSF) Product on Growth Performance, Feed Efficiency and Gut Morphology of Juvenile Nile Tilapia (*Oreochromis Niloticus*) Fed Different UK Lupin Meal Cultivars. Aquaculture (2020) 523:735192. doi: 10.1016/j.aquaculture.2020.735192

[17] Egerton S Culloty S Whooley J Stanton C Ross RP. The Gut Microbiota of Marine Fish. Front Microbiol (2018) 9:873. doi: 10.3389/fmicb.2018.00873 29780377 PMC5946678

[18] Løkka G Koppang EO. Antigen Sampling in the Fish Intestine. Dev Comp Immunol (2016) 64:138–49. doi: 10.1016/j.dci.2016.02.014 26872546

[19] Wang Z Du J Lam SH Mathavan S Matsudaira P Gong Z. Morphological and Molecular Evidence for Functional Organization Along the Rostrocaudal Axis of the Adult Zebrafish Intestine. BMC Genomics (2010) 11(1):1–13. doi: 10.1186/1471-2164-11-392 20565988 PMC2996925

[20] Løkka G Austbø L Falk K Bjerkås I Koppang EO. Intestinal Morphology of the Wild Atlantic Salmon (*Salmo Salar*). J Morphol (2013) 274(8):859–76. doi: 10.1002/jmor.20142 23520065

[21] Løkka G Austbø L Falk K Bromage E Fjelldal PG Hansen T. Immune Parameters in the Intestine of Wild and Reared Unvaccinated and Vaccinated Atlantic Salmon (*Salmo Salar* L.). Dev Comp Immunol (2014) 47(1):6–16. doi: 10.1016/j.dci.2014.06.009 24968078

[22] MacDonald NL Stark JR Austin B. Bacterial Microflora in the Gastro-Intestinal Tract of Dover Sole (*Solea Solea* L.), With Emphasis on the Possible Role of Bacteria in the Nutrition of the Host. FEMS Microbiol Lett (1986) 35(1):107–11. doi: 10.1111/j.1574-6968.1986.tb01508.x

[23] Abelli L Picchietti S Romano N Mastrolia L Scapigliati G. Immunohistochemistry of Gut-Associated Lymphoid Tissue of the Sea Bass *Dicentrarchus Labrax* (L.). Fish Shellfish Immunol (1997) 7(4):235–45. doi: 10.1006/fsim.1996.0079

[24] Wang HY Wang YJ Wang QY Xue CH Sun M. Purification and Characterization of Stomach Protease From the Turbot (*Scophthalmus Maximus* L.). Fish Physiol Biochem (2006) 32(2):179–88. doi: 10.1007/s10695-006-0010-9

[25] Chen K Zhao F Ouyang G Shi Z Ma L Wang B. Molecular Characterization and Expression Analysis of Tf_TLR4 and Tf_TRIL in Yellow Catfish Tachysurus Fulvidraco Responding to *Edwardsiella Ictaluri* Challenge. Int J Biol Macromol (2021) 167:746–55. doi: 10.1016/j.ijbiomac.2020.11.196 33278446

[26] Yang HL Sun YZ Ma RL Ye JD. PCR-DGGE Analysis of the Autochthonous Gut Microbiota of Grouper *Epinephelus Coioides* Following Probiotic *Bacillus Clausii* Administration. Aquac Res (2012) 43(4):489–97. doi: 10.1111/j.1365-2109.2011.02852.x

[27] Fuglem B Jirillo E Bjerkås I Kiyono H Nochi T Yuki Y. Antigen-Sampling Cells in the Salmonid Intestinal Epithelium. Dev Comp Immunol (2010) 34(7):768–74. doi: 10.1016/j.dci.2010.02.007 20178814

[28] Le HT Shao X Krogdahl Å Kortner TM Lein I Kousoulaki K. Intestinal Function of the Stomachless Fish, Ballan Wrasse (*Labrus Bergylta*). Front Mar Sci (2019) 6:140. doi: 10.3389/fmars.2019.00140

[29] Salinas I. The Mucosal Immune System of Teleost Fish. Biology (2015) 4(3):525–39. doi: 10.3390/biology4030525 PMC4588148 26274978

[30] Sommer F Bäckhed F. The Gut Microbiota—Masters of Host Development and Physiology. Nat Rev Microbiol (2013) 11(4):227–38. doi: 10.1038/nrmicro2974 23435359

[31] Brugman S. The Zebrafish as a Model to Study Intestinal Inflammation. Dev Comp Immunol (2016) 64:82–92. doi: 10.1016/j.dci.2016.02.020 26902932

[32] Firmino JP Galindo-Villegas J Reyes-López FE Gisbert E. Phytogenic Bioactive Compounds Shape Fish Mucosal Immunity. Front Immunol (2021) 12. doi: 10.3389/fimmu.2021.695973 PMC8252966 34220858

[33] Park Y Zhang Q Wiegertjes GF Fernandes JM Kiron V. Adherent Intestinal Cells From Atlantic Salmon Show Phagocytic Ability and Express Macrophage-Specific Genes. Front Cell Dev Biol (2020) 8. doi: 10.3389/fcell.2020.580848 PMC7593592 33178695

[34] Zhang YA Salinas I Li J Parra D Bjork S Xu Z. IgT, A Primitive Immunoglobulin Class Specialized in Mucosal Immunity. Nat Immunol (2010) 11(9):827–35. doi: 10.1038/ni.1913 PMC3459821 20676094

[35] Picchietti S Guerra L Bertoni F Randelli E Belardinelli MC Buonocore F. Intestinal T Cells of *Dicentrarchus Labrax* (L.): Gene Expression and Functional Studies. Fish Shellfish Immunol (2011) 30(2):609–17. doi: 10.1016/j.fsi.2010.12.006 21168509

[36] Ji JF Hu CB Shao T Fan DD Zhang N Lin AF. Differential Immune Responses of Immunoglobulin Z Subclass Members in Antibacterial Immunity in a Zebrafish Model. Immunology (2021) 162(1):105–20. doi: 10.1111/imm.13269 PMC7730029 32979273

[37] Hu YL Xiang LX Shao JZ. Identification and Characterization of a Novel Immunoglobulin Z Isotype in Zebrafish: Implications for a Distinct B Cell Receptor in Lower Vertebrates. Mol Immunol (2010) 47(4):738–46. doi: 10.1016/j.molimm.2009.10.010 19931913

[38] Picchietti S Buonocore F Guerra L Belardinelli MC De Wolf T Couto A. Molecular and Cellular Characterization of European Sea Bass CD3ϵ+ T Lymphocytes and Their Modulation by Microalgal Feed Supplementation. Cell Tissue Res (2021) 384(1):149–65. doi: 10.1007/s00441-020-03347-x 33433686

[39] Buonocore F Castro R Randelli E Lefranc MP Six A Kuhl H. Diversity, Molecular Characterization and Expression of T Cell Receptor γ in a Teleost Fish, the Sea Bass (*Dicentrarchus Labrax*, L). PloS One (2012) 7(10):e47957. doi: 10.1371/journal.pone.0047957 23133531 PMC3485050

[40] Wan F Hu CB Ma JX Gao K Xiang LX Shao JZ. Characterization of γδ T Cells From Zebrafish Provides Insights Into Their Important Role in Adaptive Humoral Immunity. Front Immunol (2017) 7:675. doi: 10.3389/fimmu.2016.00675 28119690 PMC5220103

[41] Graves CL Chen A Kwon V Shiau CE. Zebrafish Harbor Diverse Intestinal Macrophage Populations Including a Subset Intimately Associated With Enteric Neural Processes. iScience (2021) 24(6):102496. doi: 10.1016/j.isci.2021.102496 34142024 PMC8185245

[42] Earley AM Graves CL Shiau CE. Critical Role for a Subset of Intestinal Macrophages in Shaping Gut Microbiota in Adult Zebrafish. Cell Rep (2018) 25(2):424–36. doi: 10.1016/j.celrep.2018.09.025 PMC6245655 30304682

[43] Wiegertjes GF Wentzel AS Spaink HP Elks PM Fink IR. Polarization of Immune Responses in Fish: The ‘Macrophages First’ Point of View. Mol Immunol (2016) 69:146–56. doi: 10.1016/j.molimm.2015.09.026 26471699

[44] Stosik M Tokarz-Deptuła B Deptuła W. Immunological Memory in Teleost Fish. Fish Shellfish Immunol (2021) 115:95–103. doi: 10.1016/j.fsi.2021.05.022 34058353

[45] Havixbeck JJ Barreda DR. Neutrophil Development, Migration, and Function in Teleost Fish. Biology (2015) 4(4):715–34. doi: 10.3390/biology4040715 PMC4690015 26561837

[46] Reite OB. Mast Cells/Eosinophilic Granule Cells of Teleostean Fish: A Review Focusing on Staining Properties and Functional Responses. Fish Shellfish Immunol (1998) 8(7):489–513. doi: 10.1006/fsim.1998.0162

[47] Dezfuli BS Lui A Pironi F Manera M Shinn AP Lorenzoni M. Cell Types and Structures Involved in Tench, *Tinca Tinca* (L.), Defence Mechanisms Against a Systemic Digenean Infection. J Fish Dis (2013) 36(6):577–85. doi: 10.1111/jfd.12049 23294469

[48] Attaya A Secombes CJ Wang T. Effective Isolation of GALT Cells: Insights Into the Intestine Immune Response of Rainbow Trout (*Oncorhynchus Mykiss*) to Different Bacterin Vaccine Preparations. Fish Shellfish Immunol (2020) 105:378–92. doi: 10.1016/j.fsi.2020.06.051 32615166

[49] Parra D Korytář T Takizawa F Sunyer JO. B Cells and Their Role in the Teleost Gut. Dev Comp Immunol (2016) 64:150–66. doi: 10.1016/j.dci.2016.03.013 PMC5125549 26995768

[50] Ballesteros NA Castro R Abos B Rodríguez Saint-Jean SS Pérez-Prieto SI Tafalla C. The Pyloric Caeca Area Is a Major Site for IgM+ and IgT+ B Cell Recruitment in Response to Oral Vaccination in Rainbow Trout. PloS One (2013) 8(6):e66118. doi: 10.1371/journal.pone.0066118 23785475 PMC3681912

[51] Øverland HS Pettersen EF Rønneseth A Wergeland HI. Phagocytosis by B-Cells and Neutrophils in Atlantic Salmon (*Salmo Salar* L.) and Atlantic Cod (*Gadus Morhua* L.). Fish Shellfish Immunol (2010) 28(1):193–204. doi: 10.1016/j.fsi.2009.10.021 19874896

[52] Wu L Qin Z Liu H Lin L Ye J Li J. Recent Advances on Phagocytic B Cells in Teleost Fish. Front Immunol (2020) 11:824. doi: 10.3389/fimmu.2020.00824 32536909 PMC7267004

[53] Scapigliati G Fausto AM Picchietti S. Fish Lymphocytes: An Evolutionary Equivalent of Mammalian Innate-Like Lymphocytes? Front Immunol (2018) 9:971. doi: 10.3389/fimmu.2018.00971 29867952 PMC5949566

[54] Semple SL Dixon B. Salmonid Antibacterial Immunity: An Aquaculture Perspective. Biology (2020) 9(10):331. doi: 10.3390/biology9100331 PMC7599743 33050557

[55] Xu Z Takizawa F Parra D Gómez D von Gersdorff Jørgensen L LaPatra SE. Mucosal Immunoglobulins at Respiratory Surfaces Mark an Ancient Association That Predates the Emergence of Tetrapods. Nat Commun (2016) 7(1):1–14. doi: 10.1038/ncomms10728 PMC4754351 26869478

[56] Perdiguero P Martín-Martín A Benedicenti O Díaz-Rosales P Morel E Muñoz-Atienza E. Teleost IgD+ IgM– B Cells Mount Clonally Expanded and Mildly Mutated Intestinal IgD Responses in the Absence of Lymphoid Follicles. Cell Rep (2019) 29(13):4223–35. doi: 10.1016/j.celrep.2019.11.101 PMC6941218 31875534

[57] Edholm ES Bengtén E Stafford JL Sahoo M Taylor EB Miller NW. Identification of Two IgD+ B Cell Populations in Channel Catfish, Ictalurus Punctatus. J Immunol Res (2010) 185(7):4082–94. doi: 10.4049/jimmunol.1000631 20817869

[58] Kong WG Yu YY Dong S Huang ZY Ding LG Cao JF. Pharyngeal Immunity in Early Vertebrates Provides Functional and Evolutionary Insight Into Mucosal Homeostasis. J Immunol (2019) 203(11):3054–67. doi: 10.4049/jimmunol.1900863 PMC6859377 31645417

[59] Yu YY Kong WG Xu HY Huang ZY Zhang XT Ding LG. Convergent Evolution of Mucosal Immune Responses at the Buccal Cavity of Teleost Fish. iScience (2019) 19:821–35. doi: 10.1016/j.isci.2019.08.034 PMC6734174 31499339

[60] Boschi I Randelli E Buonocore F Casani D Bernini C Fausto AM. Transcription of T Cell-Related Genes in Teleost Fish, and the European Sea Bass (*Dicentrarchus Labrax*) as a Model. Fish Shellfish Immunol (2011) 31(5):655–62. doi: 10.1016/j.fsi.2010.10.001 20950688

[61] Yamasaki M Araki K Nakanishi T Nakayasu C Yamamoto A`. Role of CD4+ and CD8α+ T Cells in Protective Immunity Against Edwardsiella Tarda Infection of Ginbuna Crucian Carp, *Carassius Auratus Langsdorfii* . Fish Shellfish Immunol (2014) 36(1):299–304. doi: 10.1016/j.fsi.2013.11.016 24316500

[62] Somamoto T Kondo M Nakanishi T Nakao M. Helper Function of CD4+ Lymphocytes in Antiviral Immunity in Ginbuna Crucian Carp, Carassius Auratus Langsdorfii. Dev Comp Immunol (2014) 44(1):111–5. doi: 10.1016/j.dci.2013.12.008 24342571

[63] Ashfaq H El-Matbouli M Soliman H. Identification and Molecular Characterization of CD4 Genes in Brown Trout (*Salmo Trutta*). Dev Comp Immunol (2020) 107:103663. doi: 10.1016/j.dci.2020.103663 32114249

[64] Ashfaq H Soliman H Fajmann S Sexl V El-Matbouli M Saleh M. Kinetics of CD4-1+ Lymphocytes in Brown Trout After Exposure to Viral Haemorrhagic Septicaemia Virus. J Fish Dis (2021) 44:1553–62. doi: 10.1111/jfd.13476 34160839

[65] Kato G Goto K Akune I Aoka S Kondo H Hirono I. CD4 and CD8 Homologues in Japanese Flounder, *Paralichthys Olivaceus*: Differences in the Expressions and Localizations of CD4-1, CD4-2, Cd8α and CD8β. Dev Comp Immunol (2013) 39(3):293–301. doi: 10.1016/j.dci.2012.09.004 23089138

[66] Jung JW Lee AR Kim J Kim YR Lazarte JMS Lee JS. Elucidating the Functional Roles of Helper and Cytotoxic T Cells in the Cell-Mediated Immune Responses of Olive Flounder (*Paralichthys Olivaceus*). Int J Mol Sci (2021) 22(2):847. doi: 10.3390/ijms22020847 PMC7829854 33467734

[67] Dee CT Nagaraju RT Athanasiadis EI Gray C Del Ama LF Johnston SA. CD4-Transgenic Zebrafish Reveal Tissue-Resident Th2-And Regulatory T Cell–Like Populations and Diverse Mononuclear Phagocytes. J Immunol Res (2016) 197(9):3520–30. doi: 10.4049/jimmunol.1600959 PMC5073357 27694495

[68] Quintana FJ Iglesias AH Farez MF Caccamo M Burns EJ Kassam N. Adaptive Autoimmunity and Foxp3-Based Immunoregulation in Zebrafish. PloS One (2010) 5(3):e9478. doi: 10.1371/journal.pone.0009478 20221429 PMC2832694

[69] Kikuchi K. New Function of Zebrafish Regulatory T Cells in Organ Regeneration. Curr Opin Immunol (2020) 63:7–13. doi: 10.1016/j.coi.2019.10.001 31765917

[70] Stroband HWJ Van Der Veen FH. Localization of Protein Absorption During Transport of Food in the Intestine of the Grasscarp, *Ctenopharyngodon Idella* (Val.). J Exp Zool (1981) 218(2):149–56. doi: 10.1002/jez.1402180207

[71] Rombout JHWM Lamers CHJ Helfrich MH Dekker A Taverne-Thiele JJ. Uptake and Transport of Intact Macromolecules in the Intestinal Epithelium of Carp (*Cyprinus Carpio* L.) and the Possible Immunological Implications. Cell Tissue Res (1985) 239(3):519–30. doi: 10.1007/BF00219230 3986879

[72] Rombout JHWM Van den Berg AA. Immunological Importance of the Second Gut Segment of Carp. I. Uptake and Processing of Antigens by Epithelial Cells and Macrophages. J Fish Biol (1989) 35(1):13–22. doi: 10.1111/j.1095-8649.1989.tb03388.x

[73] Amthauer R Tobar L Molina H Concha M Villanueva J. Horseradish Peroxidase Binding to Intestinal Brush-Border Membranes of *Cyprinus Carpio.* Identification of a Putative Receptor. J Cell Biochem (2001) 80(2):274–84. doi: 10.1002/1097-4644(20010201)80:2<274::AID-JCB170>3.0.CO;2-A 11074599

[74] Ringø E Olsen RE Vecino JG Wadsworth S Song SK. Use of Immunostimulants and Nucleotides in Aquaculture: A Review. J Mar Sci Res Dev (2012) 2(1):104. doi: 10.4172/2155-9910.1000104

[75] Georgopoulou U Dabrowski K Sire MF Vernier JM. Absorption of Intact Proteins by the Intestinal Epithelium of Trout, *Salmo Gairdneri* . Cell Tissue Res (1988) 251(1):145–52. doi: 10.1007/BF00215459 3277712

[76] Rombout JH Yang G Kiron V. Adaptive Immune Responses at Mucosal Surfaces of Teleost Fish. Fish Shellfish Immunol (2014) 40(2):634–43. doi: 10.1016/j.fsi.2014.08.020 25150451

[77] Somamoto T Nakanishi T. Mucosal Delivery of Fish Vaccines: Local and Systemic Immunity Following Mucosal Immunisations. Fish Shellfish Immunol (2020) 99:199–207. doi: 10.1016/j.fsi.2020.01.005 31911291

[78] Bassity E Clark TG. Functional Identification of Dendritic Cells in the Teleost Model, Rainbow Trout (*Oncorhynchus Mykiss*). PloS One (2012) 7(3):e33196. doi: 10.1371/journal.pone.0033196 22427987 PMC3299753

[79] Soleto I Granja AG Simón R Morel E Díaz-Rosales P Tafalla C. Identification of CD8α+ Dendritic Cells in Rainbow Trout (*Oncorhynchus Mykiss*) Intestine. Fish Shellfish Immunol (2019) 89:309–18. doi: 10.1016/j.fsi.2019.04.001 PMC6525785 30959183

[80] Lugo-Villarino G Balla KM Stachura DL Bañuelos K Werneck MB Traver D. Identification of Dendritic Antigen-Presenting Cells in the Zebrafish. PNAS (2010) 107(36):15850–5. doi: 10.1073/pnas.1000494107 PMC2936643 20733076

[81] Kato G Miyazawa H Nakayama Y Ikari Y Kondo H Yamaguchi T. A Novel Antigen-Sampling Cell in the Teleost Gill Epithelium With the Potential for Direct Antigen Presentation in Mucosal Tissue. Front Immunol (2018) 9:2116. doi: 10.3389/fimmu.2018.02116 30294324 PMC6158387

[82] Vigneulle M Laurencin FB. Uptake of *Vibrio Anguillarum* Bacterin in the Posterior Intestine of Rainbow Trout *Oncorhynchus Mykiss*, Sea Bass *Dicentrarchus Labrax* and Turbot *Scophthalmus Maximus* After Oral Administration or Anal Intubation. Dis Aquat Organ (1991) 11(2):85–92. doi: 10.3354/dao011085

[83] Løkka G Falk K Austbø L Koppang EO. Uptake of Yeast Cells in the Atlantic Salmon (*Salmo Salar* L.) Intestine. Dev Comp Immunol (2014) 47(1):77–80. doi: 10.1016/j.dci.2014.07.005 25020196

[84] Khimmakthong U Deshmukh S Chettri JK Bojesen AM Kania PW Dalsgaard I. Tissue Specific Uptake of Inactivated and Live Yersinia Ruckeri in Rainbow Trout (*Oncorhynchus Mykiss*): Visualization by Immunohistochemistry and *in Situ* Hybridization. Microb Pathog (2013) 59:33–41. doi: 10.1016/j.micpath.2013.03.001 23583292

[85] Lie KK Tørresen OK Solbakken MH Rønnestad I Tooming-Klunderud A Nederbragt AJ. Loss of Stomach, Loss of Appetite? Sequencing of the Ballan Wrasse (*Labrus Bergylta*) Genome and Intestinal Transcriptomic Profiling Illuminate the Evolution of Loss of Stomach Function in Fish. BMC Genomics (2018) 19(1):1–17. doi: 10.1186/s12864-018-4570-8 29510660 PMC5840709

[86] Inami M Taverne-Thiele AJ Schrøder MB Kiron V Rombout JH. Immunological Differences in Intestine and Rectum of Atlantic Cod (*Gadus Morhua* L.). Fish Shellfish Immunol (2009) 26(5):751–9. doi: 10.1016/j.fsi.2009.03.007 19332137

[87] Ordás MC González-Torres L Arense P Heavyside R Zarza C Tafalla C. Analysis of Immunostimulatory Responses and Immune Tolerance to β-Glucans in Rainbow Trout Cell Lines. Aquaculture (2021) 541:736805. doi: 10.1016/j.aquaculture.2021.736805

[88] Løvmo SD Speth MT Repnik U Koppang EO Griffiths GW Hildahl JP. Translocation of Nanoparticles and *Mycobacterium Marinum* Across the Intestinal Epithelium in Zebrafish and the Role of the Mucosal Immune System. Dev Comp Immunol (2017) 67:508–18. doi: 10.1016/j.dci.2016.06.016 27343826

[89] Sato A Okamoto N. Oral and Anal Immunisation With Alloantigen Induces Active Cell-Mediated Cytotoxic Responses in Carp. Fish Shellfish Immunol (2007) 1(23):237–41. doi: 10.1016/j.fsi.2006.09.010 17113308

[90] Yamaguchi T Takizawa F Furihata M Soto-Lampe V Dijkstra JM Fischer U. Teleost Cytotoxic T Cells. Fish Shellfish Immunol (2019) 95:422–39. doi: 10.1016/j.fsi.2019.10.041 31669897

[91] Douxfils J Fierro-Castro C Mandiki SNM Emile W Tort L Kestemont P. Dietary β-Glucans Differentially Modulate Immune and Stress-Related Gene Expression in Lymphoid Organs From Healthy and *Aeromonas Hydrophila*-Infected Rainbow Trout (*Oncorhynchus Mykiss*). Fish Shellfish Immunol (2017) 63:285–96. doi: 10.1016/j.fsi.2017.02.027 28232282

[92] Mutoloki S Munang’andu HM Evensen Ø. Oral Vaccination of Fish–Antigen Preparations, Uptake, and Immune Induction. Front Immunol (2015) 6:519. doi: 10.3389/fimmu.2015.00519 26539192 PMC4610203

[93] Li Y Kortner TM Chikwati EM Munang'andu HM Lock EJ Krogdahl Å. Gut Health and Vaccination Response in Pre-Smolt Atlantic Salmon (*Salmo Salar*) Fed Black Soldier Fly (*Hermetia Illucens*) Larvae Meal. Fish Shellfish Immunol (2019) 86:1106–13. doi: 10.1016/j.fsi.2018.12.057 30590165

[94] Chen L Klaric G Wadsworth S Jayasinghe S Kuo TY Evensen Ø. Augmentation of the Antibody Response of Atlantic Salmon by Oral Administration of Alginate-Encapsulated IPNV Antigens. PloS One (2014) 9(10):e109337. doi: 10.1371/journal.pone.0109337 25310804 PMC4195674

[95] Anderson DP. Immunostimulants, Adjuvants, and Vaccine Carriers in Fish: Applications to Aquaculture. Annu Rev Fish Dis (1992) 2:281–307. doi: 10.1016/0959-8030(92)90067-8

[96] Bricknell I Dalmo RA. The Use of Immunostimulants in Fish Larval Aquaculture. Fish Shellfish Immunol (2005) 19:457–72. doi: 10.1016/j.fsi.2005.03.008 15890531

[97] Chang J. Medicinal Herbs: Drugs or Dietary Supplements? Biochem Pharmacol (2000) 59:211–9. doi: 10.1016/S0006-2952(99)00243-9 10609549

[98] Varijakzhan D Chong CM Abushelaibi A Lai KS Lim SHE. Middle Eastern Plant Extracts: An Alternative to Modern Medicine Problems. Molecules (2020) 25(5):1126. doi: 10.3390/molecules25051126 PMC7179161 32138245

[99] Yap PSX Yusoff K Lim SHE Chong CM Lai KS. Membrane Disruption Properties of Essential Oils—A Double-Edged Sword? Processes (2021) 9(4):595. doi: 10.3390/pr9040595

[100] Liu B Xie J Ge X Xu P Wang A He Y. Effects of Anthraquinone Extract From Rheum Officinale Bail on the Growth Performance and Physiological Responses of Macrobrachium Rosenbergii Under High Temperature Stress. Fish Shellfish Immunol (2010) 29:49–57. doi: 10.1016/j.fsi.2010.02.018 20219682

[101] Tan X Sun Z Chen S Chen S Huang Z Zhou C. Effects of Dietary Dandelion Extracts on Growth Performance, Body Composition, Plasma Biochemical Parameters, Immune Responses and Disease Resistance of Juvenile Golden Pompano *Trachinotus Ovatus* . Fish Shellfish Immunol (2017) 66:198–206. doi: 10.1016/j.fsi.2017.05.028 28499965

[102] Eirna-Liza N Hassim HA Min CC Syukri F Karim M. The Duration of Protection Conferred by Garlic on African Catfish (*Clarias Gariepinus*) Against Aeromonas Hydrophila. J Aquac Res Dev (2018) 9(552):2. doi: 10.4172/2155-9546.1000552

[103] Chong CM Murthy AG Choy CY Lai KS. Phytotherapy in Aquaculture: Integration of Endogenous Application With Science. J Environ Biol (2020) 41:1204–14. doi: 10.22438/jeb/41/5(SI)/MS_12

[104] Reverter M Bontemps N Lecchini D Banaigs B Sasal P. Use of Plant Extracts in Fish Aquaculture as an Alternative to Chemotherapy: Current Status and Future Perspectives. Aquaculture (2014) 433:50–61. doi: 10.1016/j.aquaculture.2014.05.048

[105] Liu B Ge X Xie J Xu P He Y Cui Y. Effects of Anthraquinone Extract From Rheum Officinale Bail on the Physiological Responses and HSP70 Gene Expression of Megalobrama Amblycephala Under *Aeromonas Hydrophila* Infection. Fish Shellfish Immunol (2012) 32:1–7. doi: 10.1016/j.fsi.2011.02.015 21362482

[106] Varijakzhan D Yang SK Chong CM Akseer R Alhosani MS Thomas W. Essential Oils as Potential Antimicrobial Agents. In: Panwar H Sharma C Lichtfouse E, editors. Sustainable Agriculture Reviews, vol. 49. Cham: Springer (2021). p. 93–122.

[107] Yin G Jeney G Stromájer-Rácz T Xu P Jun X. Effect of Two Chinese Herbs (*Astragalus Radix* and Scutellaria Radix) on non-Specific Immune Response of Tilapia, Oreochromis Niloticus. Aquaculture (2006) 253:39–47. doi: 10.1016/j.aquaculture.2005.06.038

[108] Galina J Yin G Ardo L Jeney Z. The Use of Immunostimulating Herbs in Fish. An Overview of Research. Fish Physiol Biochem (2009) 35:669–76. doi: 10.1007/s10695-009-9304-z 19277888

[109] Yin G Ardo L Thompson KD Adams A Jeney Z Jeney G. Chinese Herbs (*Astragalus Radix* and Ganoderma Lucidum) Enhance Immune Response of Carp, Cyprinus Carpio, and Protection Against *Aeromonas Hydrophila* . Fish Shellfish Immunol (2009) 26:140–5. doi: 10.1016/j.fsi.2008.08.015 18817878

[110] Wu YS Lee MC Huang CT Kung TC Huang CY Nan FH. Effects of Traditional Medical Herbs "Minor Bupleurum Decoction" on the non-Specific Immune Responses of White Shrimp (*Litopenaeus Vannamei*). Fish Shellfish Immunol (2017) 64:218–25. doi: 10.1016/j.fsi.2017.03.018 28288911

[111] Baba E Acar Ü Yılmaz S Zemheri F Ergün S. Dietary Olive Leaf (*Olea Europea* L.) Extract Alters Some Immune Gene Expression Levels and Disease Resistance to Yersinia Ruckeri Infection in Rainbow Trout *Oncorhynchus Mykiss* . Fish Shellfish Immunol (2018) 79:28–33. doi: 10.1016/j.fsi.2018.04.063 29733961

[112] Zahran E Abd El-Gawad EA Risha E. Dietary Withania Sominefera Root Confers Protective and Immunotherapeutic Effects Against *Aeromonas Hydrophila* Infection in Nile Tilapia (*Oreochromis Niloticus*). Fish Shellfish Immunol (2018) 80:641–50. doi: 10.1016/j.fsi.2018.06.009 29886140

[113] Zemheri-Navruz F Acar Ü Yılmaz S. Dietary Supplementation of Olive Leaf Extract Increases Haematological, Serum Biochemical Parameters and Immune Related Genes Expression Level in Common Carp (*Cyprinus Carpio*) Juveniles. Fish Shellfish Immunol (2019) 89:672–6. doi: 10.1016/j.fsi.2019.04.037 30991150

[114] Harikrishnan R Devi G Paray BA Al-Sadoon MK Al-Mfarij AR Van Doan H. Effect of Cassic Acid on Immunity and Immune-Reproductive Genes Transcription in Clarias Gariepinus Against *Edwardsiella Tarda* . Fish Shellfish Immunol (2020) 99:331–41. doi: 10.1016/j.fsi.2020.02.037 32084536

[115] Yakubu Y Talba AM Chong CM Ismail IS Shaari K. Effect of Terminalia Catappa Methanol Leaf Extract on Nonspecific Innate Immune Responses and Disease Resistance of Red Hybrid Tilapia Against *Streptococcus Agalactiae* . Aquac Rep (2020) 18:100555. doi: 10.1016/j.aqrep.2020.100555

[116] Hoseinifar SH Sohrabi A Paknejad H Jafari V Paolucci M Van Doan H. Enrichment of Common Carp (*Cyprinus Carpio*) Fingerlings Diet With *Psidium Guajava*: The Effects on Cutaneous Mucosal and Serum Immune Parameters and Immune Related Genes Expression. Fish Shellfish Immunol (2019) 86:688–94. doi: 10.1016/j.fsi.2018.12.001 30521968

[117] Hoseinifar SH Shakouri M Doan HV Shafiei S Yousefi M Raeisi M. Dietary Supplementation of Lemon Verbena (*Aloysia Citrodora*) Improved Immunity, Immune-Related Genes Expression and Antioxidant Enzymes in Rainbow Trout (*Oncorrhyncus Mykiss*). Fish Shellfish Immunol (2020) 99:379–85. doi: 10.1016/j.fsi.2020.02.006 32032763

[118] Ahmed SAA Abd El-Rahman GI Behairy A Beheiry RR Hendam BM Alsubaie FM. Influence of Feeding Quinoa (*Chenopodium Quinoa*) Seeds and Prickly Pear Fruit (*Opuntia Ficus Indica*) Peel on the Immune Response and Resistance to Aeromonas Sobria Infection in Nile Tilapia (*Oreochromis Niloticus*). Animals (2020) 10:2266. doi: 10.3390/ani10122266 PMC7760620 33271917

[119] Giri SS Sukumaran V Park SC. Effects of Bioactive Substance From Turmeric on Growth, Skin Mucosal Immunity and Antioxidant Factors in Common Carp, Cyprinus Carpio. Fish Shellfish Immunol (2019) 92:612–20. doi: 10.1016/j.fsi.2019.06.053 31265909

[120] Meng X Hu W Wu S Zhu Z Lu R Yang G. Chinese Yam Peel Enhances the Immunity of the Common Carp (*Cyprinus Carpio* L.) by Improving the Gut Defence Barrier and Modulating the Intestinal Microflora. Fish Shellfish Immunol (2019) 95:528–37. doi: 10.1016/j.fsi.2019.10.066 31678187

[121] Safari R Hoseinifar SH Nejadmoghadam S Jafar A. Transciptomic Study of Mucosal Immune, Antioxidant and Growth Related Genes and non-Specific Immune Response of Common Carp (*Cyprinus Carpio*) Fed Dietary Ferula (*Ferula Assafoetida*). Fish Shellfish Immunol (2016) 55:242–8. doi: 10.1016/j.fsi.2016.05.038 27241284

[122] Tan X Sun Z Ye C. Dietary Ginkgo Biloba Leaf Extracts Supplementation Improved Immunity and Intestinal Morphology, Antioxidant Ability and Tight Junction Proteins mRNA Expression of Hybrid Groupers (*Epinephelus Lanceolatus* ♂ × *Epinephelus Fuscoguttatus* ♀) Fed High Lipid Diets. Fish Shellfish Immunol (2020) 98:611–8. doi: 10.1016/j.fsi.2019.09.034 31533081

[123] Bao L Chen Y Li H Zhang J Wu P Ye K. Dietary Ginkgo Biloba Leaf Extract Alters Immune-Related Gene Expression and Disease Resistance to *Aeromonas Hydrophila* in Common Carp *Cyprinus Carpio* . Fish Shellfish Immunol (2019) 94:810–8. doi: 10.1016/j.fsi.2019.09.056 31546037

[124] Espinosa C García Beltrán JM Messina CM Esteban MÁ. Effect of Jasonia Glutinosa on Immune and Oxidative Status of Gilthead Seabream (*Sparus Aurata* L.). Fish Shellfish Immunol (2020) 100:58–69. doi: 10.1016/j.fsi.2020.02.068 32145448

[125] Terzi E Kucukkosker B Bilen S Kenanoğlu O Corum O Özbek M. A Novel Herbal Immunostimulant for Rainbow Trout (Oncorhynchus mykiss) Against Yersinia Ruckeri. Fish Shellfish Immunol (2021) 110:55–66. doi: 10.1016/j.fsi.2020.12.019 33383177

[126] Omitoyin B Ajani E Olugbenga O Bassey HE Osho F. Effect of Guava Psidium Guajava (L.) Aqueous Extract Diet on Growth Performance, Intestinal Morphology, Immune Response and Survival of Oreochromis Niloticus Challenged With Aeromonas Hydrophila. Aquac Res (2019), 1–11. doi: 10.1111/are.14068

[127] Wei L Wu P Zhou X-Q Jiang W-D Liu Y Kuang S-Y. Dietary Silymarin Supplementation Enhanced Growth Performance and Improved Intestinal Apical Junctional Complex on Juvenile Grass Carp (*Ctenopharyngodon Idella*). Aquaculture (2020) 525:735311. doi: 10.1016/j.aquaculture.2020.735311

[128] Mousavi S Sheikhzadeh N Hamidian G Mardani K Oushani AK Firouzamandi M. Changes in Rainbow Trout (*Oncorhynchus Mykiss*) Growth and Mucosal Immune Parameters After Dietary Administration of Grape (*Vitis Vinifera*) Seed Extract. Fish Physiol Biochem (2021) 47(2):547–63. doi: 10.1007/s10695-021-00930-z 33543428

[129] Zahran E El Sebaei MG Awadin W Elbahnaswy S Risha E Elseady Y. Withania Somnifera Dietary Supplementation Improves Lipid Profile, Intestinal Histomorphology in Healthy Nile Tilapia (*Oreochromis Niloticus*), and Modulates Cytokines Response to *Streptococcus* Infection. Fish Shellfish Immunol (2020) 106:133–41. doi: 10.1016/j.fsi.2020.07.056 32738514

[130] Wang L Wu D Fan Z Li H Li J Zhang Y. Effect of Yucca Schidigera Extract on the Growth Performance, Intestinal Antioxidant Status, Immune Response, and Tight Junctions of Mirror Carp (*Cyprinus Carpio*). Fish Shellfish Immunol (2020) 103:211–9. doi: 10.1016/j.fsi.2020.05.039 32422190

[131] McDole JR Wheeler LW McDonald KG Wang B Konjufca V Knoop KA. Goblet Cells Deliver Luminal Antigen to CD103 + Dendritic Cells in the Small Intestine. Nature (2012) 483:345–9. doi: 10.1038/nature10863 PMC3313460 22422267

[132] Knoop KA McDonald KG McCrate S McDole JR Newberry RD. Microbial Sensing by Goblet Cells Controls Immune Surveillance of Luminal Antigens in the Colon. Mucosal Immunol (2015) 8:198–210. doi: 10.1038/mi.2014.58 25005358 PMC4268115

[133] Knoop KA Newberry RD. Goblet Cells: Multifaceted Players in Immunity at Mucosal Surfaces. Mucosal Immunol (2018) 11:1551–7. doi: 10.1038/s41385-018-0039-y PMC8767637 29867079

[134] Dezfuli BS Pironi F Campisi M Shinn AP Giari L. The Response of Intestinal Mucous Cells to the Presence of Enteric Helminths: Their Distribution, Histochemistry and Fine Structure. J Fish Dis (2010) 33:481–8. doi: 10.1111/j.1365-2761.2010.01146.x 20298449

[135] Zou J Secombes CJ. The Function of Fish Cytokines. Biol (Basel) (2016) 5:23. doi: 10.3390/biology5020023 PMC4929537 27231948

[136] Costa MM Maehr T Diaz-Rosales P Secombes CJ Wang T. Bioactivity Studies of Rainbow Trout (*Oncorhynchus Mykiss*) Interleukin-6: Effects on Macrophage Growth and Antimicrobial Peptide Gene Expression. Mol Immunol (2011) 48:1903–16. doi: 10.1016/j.molimm.2011.05.027 21704380

[137] Kim Y-S Morgan MJ Choksi S Liu Z-G. TNF-Induced Activation of the Nox1 NADPH Oxidase and its Role in the Induction of Necrotic Cell Death. Mol Cell (2007) 26:675–87. doi: 10.1016/j.molcel.2007.04.021 17560373

[138] Baregamian N Song J Bailey CE Papaconstantinou J Evers BM Chung DH. Tumor Necrosis Factor-Alpha and Apoptosis Signal-Regulating Kinase 1 Control Reactive Oxygen Species Release, Mitochondrial Autophagy, and C-Jun N-Terminal Kinase/P38 Phosphorylation During Necrotizing Enterocolitis. Oxid Med Cell Longev (2009) 2:297–306. doi: 10.4161/oxim.2.5.9541 20716917 PMC2835918

[139] Schroder K Tschopp J. The Inflammasomes. Cell (2010) 140:821–32. doi: 10.1016/j.cell.2010.01.040 20303873

[140] Matsuzawa A Ichijo H. Redox Control of Cell Fate by MAP Kinase: Physiological Roles of ASK1-MAP Kinase Pathway in Stress Signaling. Biochim Biophys Acta (2008) 1780:1325–36. doi: 10.1016/j.bbagen.2007.12.011 18206122

[141] Marjoram L Alvers A Deerhake ME Bagwell J Mankiewicz J Cocchiaro JL. Epigenetic Control of Intestinal Barrier Function and Inflammation in Zebrafish. PNAS (2015) 112:2770. doi: 10.1073/pnas.1424089112 25730872 PMC4352795

[142] Beck BR Lee SH Kim D Park JH Lee HK Kwon S-S. A Lactococcus Lactis BFE920 Feed Vaccine Expressing a Fusion Protein Composed of the OmpA and FlgD Antigens From Edwardsiella Tarda was Significantly Better at Protecting Olive Flounder (*Paralichthys Olivaceus*) From Edwardsiellosis Than Single Antigen Vaccines. Fish Shellfish Immunol (2017) 68:19–28. doi: 10.1016/j.fsi.2017.07.004 28687358

[143] Brudeseth B Wiulsrød R Fredriksen Børge Lindmo K Løkling KE Bordevik M. Status and Future Perspectives of Vaccines for Industrialised Fin-Fish Farming. Fish Shellfish Immunol (2013) 35(6):1759–68. doi: 10.1016/j.fsi.2013.05.029 23769873

[144] Chen Y Hua X Ren X Duan K Gao S Sun J. Oral Immunization With Recombinant *Lactobacillus Casei* Displayed AHA1-CK6 and VP2 Induces Protection Against Infectious Pancreatic Necrosis in Rainbow Trout (*Oncorhynchus Mykiss*). Fish Shellfish Immunol (2020) 100:18–26. doi: 10.1016/j.fsi.2020.03.001 32142871

[145] Embregts CWE Forlenza M. Oral Vaccination of Fish: Lessons From Humans and Veterinary Species. Dev Comp Immunol (2016) 64:118–37. doi: 10.1016/j.dci.2016.03.024 27018298

[146] Adams A. Progress, Challenges and Opportunities in Fish Vaccine Development. Fish Shellfish Immunol (2019) 90:210–4. doi: 10.1016/j.fsi.2019.04.066 31039441

[147] Tafalla C Bøgwald J Dalmo RA. Adjuvants and Immunostimulants in Fish Vaccines: Current Knowledge and Future Perspectives. Fish Shellfish Immunol (2013) 35(6):1740–50. doi: 10.1016/j.fsi.2013.02.029 23507338

[148] Ma J Bruce T Jones E Cain K. A Review of Fish Vaccine Development Strategies: Conventional Methods and Modern Biotechnological Approaches. Microorganisms (2019) 7(11):569. doi: 10.3390/microorganisms7110569 PMC6920890 31744151

[149] Gonzalez-Silvera D Guardiola FA Espinosa C Chaves-Pozo E Esteban MÁ Cuesta A. Recombinant Nodavirus Vaccine Produced in Bacteria and Administered Without Purification Elicits Humoral Immunity and Protects European Sea Bass Against Infection. Fish Shellfish Immunol (2019) 88:458–63. doi: 10.1016/j.fsi.2019.03.013 30877059

[150] Han B Xu K Liu Z Ge W Shao S Li P. Oral Yeast-Based DNA Vaccine Confers Effective Protection From Aeromonas Hydrophila Infection on *Carassius Auratus* . Fish Shellfish Immunol (2019) 84:948–54. doi: 10.1016/j.fsi.2018.10.065 30445667

[151] Liu Z Wu J Ma Y Hao L Liang Z Ma J. Protective Immunity Against CyHV-3 Infection via Different Prime-Boost Vaccination Regimens Using CyHV-3 ORF131-Based DNA/protein Subunit Vaccines in Carp *Cyprinus Carpio* Var. Jian Fish Shellfish Immunol (2020) 98:342–53. doi: 10.1016/j.fsi.2020.01.034 31978531

[152] Ringø E Hoseinifar S Ghosh K Doan H Beck BR Song S. Lactic Acid Bacteria in Finfish-An Update. Front Microbiol (2018) 9:1818. doi: 10.3389/fmicb.2018.01818 30147679 PMC6096003

[153] Olmos J Acosta M Mendoza G Pitones V. Bacillus Subtilis, An Ideal Probiotic Bacterium to Shrimp and Fish Aquaculture that Increase Feed Digestibility, Prevent Microbial Diseases, and Avoid Water Pollution. Arch Microbiol (2020) 202(3):427–35. doi: 10.1007/s00203-019-01757-2 31773195

[154] Naderi-Samani M Soltani M Dadar M Taheri-Mirghaed A Zargar A Ahmadivand S. Oral Immunization of Trout Fry With Recombinant *Lactococcus Lactis* NZ3900 Expressing G Gene of Viral Hemorrhagic Septicaemia Virus (VHSV). Fish Shellfish Immunol (2020) 105:62–70. doi: 10.1016/j.fsi.2020.07.007 32645516

[155] Zhao L Tang X Sheng X Xing J Zhan W. Surface Display of Hirame Novirhabdovirus (HIRRV) G Protein in Lactococcus Lactis and its Immune Protection in Flounder (*Paralichthys Olivaceus*). Microb Cell Fact (2019) 18:142. doi: 10.1186/s12934-019-1195-9 31434565 PMC6704618

[156] Lee SH Beck BR Hwang SH Song SK. Feeding Olive Flounder (*Paralichthys Olivaceus*) With *Lactococcus Lactis* BFE920 Expressing the Fusion Antigen of *Vibrio* OmpK and FlaB Provides Protection Against Multiple *Vibrio* Pathogens: A Universal Vaccine Effect. Fish Shellfish Immunol (2021) 114:253–62. doi: 10.1016/j.fsi.2021.05.007 33979691

[157] Ze LZ Bin TY Yang YX Song NG Dong XZ Sheng NJ. Construction and Immune Efficacy of Recombinant *Lactobacillus Casei* Expressing OmpAI of *Aeromonas Veronii* C5-I as Molecular Adjuvant. Microb Pathog (2021) 156:104827. doi: 10.1016/j.micpath.2021.104827 33892129

[158] Ju A-Q Yang S-B Zhang H-P Ma X Zhang D-X Kang Y-H. Construction and Immune Efficacy of Recombinant *Lactobacillus Casei* Strains Expressing Malt From *Aeromonas Veronii* . Microb Pathog (2020) 141:103918. doi: 10.1016/j.micpath.2019.103918 31935441

[159] Zhang L Li Z Li Y Tian J Jia K Zhang D. OmpW Expressed by Recombinant *Lactobacillus Casei* Elicits Protective Immunity Against *Aeromonas Veronii* in Common Carp. Microb Pathog (2019) 133:103552. doi: 10.1016/j.micpath.2019.103552 31121269

[160] Kong Y-D Kang Y-H Tian J-X Zhang D-X Zhang L Tao L-T. Oral Immunization With Recombinant Lactobacillus Casei Expressing flaB Confers Protection Against *Aeromonas Veronii* Challenge in Common Carp, *Cyprinus Carpio* . Fish Shellfish Immunol (2019) 87:627–37. doi: 10.1016/j.fsi.2019.01.032 30708057

[161] Zhang D-X Kang Y-H Chen L Siddiqui SA Wang C-F Qian A-D. Oral Immunization With Recombinant Lactobacillus Casei Expressing OmpAI Confers Protection Against *Aeromonas Veronii* Challenge in Common Carp, *Cyprinus Carpio* . Fish Shellfish Immunol (2018) 72:552–63. doi: 10.1016/j.fsi.2017.10.043 29155272

[162] Duan K Hua X Wang Y Wang Y Chen Y Shi W. Oral Immunization With a Recombinant Lactobacillus Expressing CK6 Fused With VP2 Protein Against IPNV in Rainbow Trout (*Oncorhynchus Mykiss*). Fish Shellfish Immunol (2018) 83:223–31. doi: 10.1016/j.fsi.2018.09.034 30217507

[163] Huang X Ma Y Wang Y Niu C Liu Z Yao X. Expressing Koi Herpesvirus (KHV) ORF81 Protein Delivered by Chitosan-Alginate Capsules Is a Promising Strategy for Mass Oral Vaccination of Carps Against KHV Infection. J Virol (2021) 95(12):e00415–21. doi: 10.1128/JVI.00415-21 PMC8315992 33827944

[164] Sun R Zhang M Chen H Wei Y Ning D. Germination-Arrest Bacillus Subtilis Spores as an Oral Delivery Vehicle of Grass Carp Reovirus (GCRV) Vp7 Antigen Augment Protective Immunity in Grass Carp (*Ctenopharyngodon Idella*). Genes (2020) 11:1351. doi: 10.3390/genes11111351 PMC7696455 33202570

[165] Jiang H Bian Q Zeng W Ren P Sun H Lin Z. Oral Delivery of *Bacillus Subtilis* Spores Expressing Grass Carp Reovirus VP4 Protein Produces Protection Against Grass Carp Reovirus Infection. Fish Shellfish Immunol (2019) 84:768–80. doi: 10.1016/j.fsi.2018.10.008 30300738

[166] Yao Y-Y Chen D-D Cui Z-W Zhang X-Y Zhou Y-Y Guo X. Oral Vaccination of Tilapia Against Streptococcus Agalactiae Using *Bacillus Subtilis* Spores Expressing Sip. Fish Shellfish Immunol (2019) 86:999–1008. doi: 10.1016/j.fsi.2018.12.060 30590166

[167] Heenatigala PPM Sun Z Yang J Zhao X Hou H. Expression of LamB Vaccine Antigen in *Wolffia Globosa* (Duck Weed) Against Fish Vibriosis. Front Immunol (2020) 11:1857. doi: 10.3389/fimmu.2020.01857 32973766 PMC7468452

[168] Nakahira Y Mizuno K Yamashita H Tsuchikura M Takeuchi K Shiina T. Mass Production of Virus-Like Particles Using Chloroplast Genetic Engineering for Highly Immunogenic Oral Vaccine Against Fish Disease. Front Plant Sci (2021) 23(12):717952. doi: 10.3389/fpls.2021.717952 PMC8419230 34497627

[169] Cho HS Seo JY Park SI Kim TG Kim TJ. Oral Immunization With Recombinant Protein Antigen Expressed in Tobacco Against Fish Nervous Necrosis Virus. J Vet Med Sci (2018) 80:272–9. doi: 10.1292/jvms.16-0408 PMC5836763 29249747

[170] Loh JY Kay GL Ting ASY. Bioencapsulation and Colonization Characteristics of Lactococcus Lactis Subsp. *Lactis* CF4MRS in *Artemia Franciscana*: A Biological Approach for the Control of *Edwardsiella Tarda* in Larviculture. Mar Biotechnol (2018) 20:353–62. doi: 10.1007/s10126-018-9813-9 29654379

[171] Szatraj K Szczepankowska A Chmielewska-Jeznach M. Lactic Acid Bacteria — Promising Vaccine Vectors: Possibilities, Limitations, Doubts. J Appl Microbiol (2017) 123:325–39. doi: 10.1111/jam.13446 PMC7166332 28295939

[172] Glenting J Wessels S. Ensuring Safety of DNA Vaccines. Microb Cell Fact (2005) 4:26. doi: 10.1186/1475-2859-4-26 16144545 PMC1215512

[173] Loh JY Ting ASY. Bioencapsulation of Probiotic Lactococcus Lactis Subsp. *Lactis* on *Artemia Franciscana* Nauplii: Effects of Encapsulation Media on Nauplii Survival and Probiotic Recovery. Malays J Microbiol (2015) 11(2):121 – 127. doi: 10.21161/mjm.12314

[174] Loh JY Lai KS Lee PT Liew HJ Ting ASY. Effects of *Artemia* Nauplii Bioencapsulated With *Lactococcus Lactis* Subsp. *Lactis* CF4MRS and Sodium Alginate on Edwardsiellosis Protection and Digestive Enzyme Production in Climbing Perch Larvae, *Anabas Testudineus* (Bloc). J Appl Aquac (2021), 1–18. doi: 10.1080/10454438.2021.1935389

[175] Maiti B Shetty M Shekar M Karunasagar I. Evaluation of Two Outer Membrane Proteins, Aha1 and OmpW of Aeromonas Hydrophila as Vaccine Candidate for Common Carp. Vet Immunol Immunopathol (2012) 149(3–4):298–301. doi: 10.1016/j.vetimm.2012.07.013 22917476

[176] Knecht LD Pasini P Daunert S. Bacterial Spores as Platforms for Bioanalytical and Biomedical Applications. Anal Bioanal Chem (2011) 400:977–89. doi: 10.1007/s00216-011-4835- 21380604

[177] Hoare R Leigh W Limakom T Wongwaradechkul R Metselaar M Shinn AP. Oral Vaccination of Nile Tilapia (*Oreochromis Niloticus*) Against Francisellosis Elevates Specific Antibody Titres in Serum and Mucus. Fish Shellfish Immunol (2021) 113:86–8. doi: 10.1016/j.fsi.2021.03.019 33826937

[178] Guo M Li C. An Overview of Cytokine Used as Adjuvants in Fish: Current State and Future Trends. Rev Aquac (2020) 13:996–1014. doi: 10.1111/raq.12509

[179] Galindo-Villegas J Mulero I García-Alcazar A Muñoz I Peñalver-Mellado M Streitenberger S. Recombinant Tnfα as Oral Vaccine Adjuvant Protects European Sea Bass Against Vibriosis: Insights Into the Role of the CCL25/CCR9 Axis. Fish Shellfish Immunol (2013) 35(4):1260–71. doi: 10.1016/j.fsi.2013.07.046 23932985

[180] Mahmood N Nasir SB Hefferon K. Plant-Based Drugs and Vaccines for COVID-19. Vaccine (2020) 9:15. doi: 10.3390/vaccines9010015 PMC7823519 33396667

[181] Xiong JB Nie L Chen J. Current Understanding on the Roles of Gut Microbiota in Fish Disease and Immunity. Zool Res (2019) 40(2):70. doi: 10.24272/j.issn.2095-8137.2018.069 29976843 PMC6378566

[182] Escalas A Auguet J-C Avouac A Seguin R Gradel A Borrossi L. Ecological Specialization Within a Carnivorous Fish Family Is Supported By a Herbivorous Microbiome Shaped By a Combination of Gut Traits and Specific Diet. Front Mar Sci (2021) 8:622883. doi: 10.3389/fmars.2021.622883

[183] Frenette A Booman M Fujiki K Kales S Ryan C Gamperl AK. Antigen Presentation Genes in Gadoid Species (Haddock: Melanogrammus Aeglefinus and Atlantic Cod: Gadus Morhua) Raise Questions About Cross-Presentation Pathways and Glycosylated Beta-2- Microglobulin. Mol Immunol (2021) 129:21–31. doi: 10.1016/j.molimm.2020.11.011 33260037

[184] Chong C Low C. Synthetic antibody: Prospects in Aquaculture Biosecurity. Fish Shellfish Immunol (2019) 86:361–7. doi: 10.1016/j.fsi.2018.11.060 30502461

[185] Mashoof S Criscitiello MF. Fish Immunoglobulins. Biology (2016) 5(4):45. doi: 10.3390/biology5040045 PMC5192425 27879632

